# Tubular Injury Biomarkers to Predict CKD and Hypertension at 3 Months Post-Cisplatin in Children

**DOI:** 10.34067/KID.0000000000000448

**Published:** 2024-04-26

**Authors:** Ryan S. Huang, Kelly R. McMahon, Stella Wang, Hayton Chui, Asaf Lebel, Jasmine Lee, Vedran Cockovski, Shahrad Rod Rassekh, Kirk R. Schultz, Tom D. Blydt-Hansen, Geoffrey D.E. Cuvelier, Cherry Mammen, Maury Pinsk, Bruce C. Carleton, Ross T. Tsuyuki, Colin J.D. Ross, Ana Palijan, Michael Zappitelli, for the Applying Biomarkers to Minimize Long-Term Effects of Childhood/Adolescent Cancer Treatment (ABLE) Research Study Group

**Affiliations:** 1 Temerty Faculty of Medicine, University of Toronto, Toronto, Ontario, Canada; 2 Division of Nephrology, Department of Pediatrics, The Hospital for Sick Children, University of Toronto, Toronto, Ontario, Canada; 3 Division of Nephrology, Department of Pediatrics, Centre for Outcomes Research and Evaluation (CORE), Research Institute of the McGill University Health Centre, Montreal, Quebec, Canada; 4 Faculty of Health Sciences, Queen's University, Kingston, Ontario, Canada; 5 Division of Hematology/Oncology/Bone Marrow Transplantation, Department of Pediatrics, British Columbia Children's Hospital, University of British Columbia, Vancouver, British Columbia, Canada; 6 Division of Pediatric Nephrology, Department of Pediatrics, British Columbia Children's Hospital, University of British Columbia, Vancouver, British Columbia, Canada; 7 Department of Pediatric Hematology-Oncology-BMT, CancerCare Manitoba, Max Rady College of Medicine, University of Manitoba, Winnipeg, Manitoba, Canada; 8 Section of Pediatric Nephrology, Department of Pediatrics and Child Health, University of Manitoba, Winnipeg, Manitoba, Canada; 9 Division of Translational Therapeutics, Department of Pediatrics, BC Children's Hospital Research Institute, University of British Columbia, Vancouver, British Columbia, Canada; 10 Departments of Pharmacology and Medicine, Faculty of Medicine and Dentistry, EPICORE Centre, University of Alberta, Edmonton, Alberta, Canada; 11 Faculty of Pharmaceutical Sciences, University of British Columbia, Vancouver, British Columbia, Canada

**Keywords:** AKI, BP, CKD, cisplatin, cisplatin nephrotoxicity, hypertension, kidney disease, nephrotoxicity, pediatric nephrology, renal injury

## Abstract

**Key Points:**

- Tubular injury biomarkers are not individually strong predictors of 3-month post-cisplatin CKD.
- When combined with clinical measures, tubular injury biomarkers can predict post-therapy hypertension and identify high-risk patients.

**Background:**

Urine kidney injury biomarkers measured during cisplatin therapy may identify patients at risk of adverse subsequent kidney outcomes. We examined relationships between tubular injury biomarkers collected early (early visit [EV]: first *or s*econd cisplatin cycle) and late (late visit: last *or* second-last cisplatin cycle) during cisplatin therapy, with 3-month post-cisplatin CKD and hypertension (HTN).

**Methods:**

We analyzed data from the Applying Biomarkers to Minimize Long-Term Effects of Childhood/Adolescent Cancer Treatment Nephrotoxicity study, a 12-center prospective cohort study of 159 children receiving cisplatin. We measured urine neutrophil gelatinase-associated lipocalin (NGAL)/creatinine, kidney injury molecule-1/creatinine, tissue inhibitor of metalloproteinase-2 (TIMP-2), and insulin-like growth factor-binding protein 7 (IGFBP-7) (TIMP-2 and IGFBP-7 expressed as their product, ng/ml^2^/1000) at an EV and late visit during cisplatin therapy with preinfusion, postinfusion, and hospital discharge sampling. Area under the curve (AUC) was calculated for biomarkers to detect 3-month post-cisplatin CKD (Kidney Disease Improving Global Outcomes guidelines: low eGFR or elevated urine albumin-to-creatinine ratio for age) and HTN (three BPs; per American Academy of Pediatrics guidelines).

**Results:**

At median follow-up of 90 days, 52 of 118 patients (44%) and 17 of 125 patients (14%) developed CKD and HTN, respectively. Biomarker prediction for 3-month CKD was low to modest; NGAL combined with kidney injury molecule-1 at EV discharge yielded the highest AUC (0.67; 95% confidence interval, 0.57 to 0.77). Biomarker prediction of 3-month HTN was stronger, but modest; the highest AUC was from combining EV preinfusion NGAL and TIMP-2×IGFBP-7 (0.71; 95% confidence interval, 0.62 to 0.80). When EV preinfusion NGAL and TIMP-2×IGFBP-7 were added to the 3-month HTN clinical predictive model, AUCs increased from 0.81 (0.72 to 0.91) to 0.89 (0.83 to 0.95) (*P* < 0.05).

**Conclusions:**

Tubular injury biomarkers we studied were individually not strong predictors of 3-month post-cisplatin kidney outcomes. Adding biomarkers to existing clinical prediction models may help predict post-therapy HTN and identify higher kidney-risk patients.

## Visual Abstract

**Figure:**
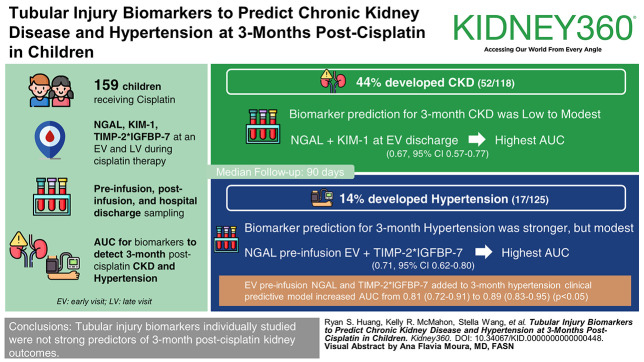

## Introduction

Cisplatin is a highly effective chemotherapeutic agent used to treat many pediatric cancers.^1,2^ Cisplatin nephrotoxicity manifesting as AKI occurs in 20%–50% of children.^3–5^ AKI is known to increase risk of CKD and hypertension (HTN).^6,7^ Currently, the ability to predict which children receiving cisplatin will develop long-term kidney abnormalities and may benefit from intensive kidney health follow-up remains limited.

Novel urine AKI biomarkers include proteins reflecting structural kidney injury. Because biomarkers of acute tubular injury indicate kidney cell injury, they may be more useful than, or enhance, traditional markers of AKI (serum creatinine [SCr]; urine output) to predict later CKD or HTN.^8,9^ Neutrophil gelatinase-associated lipocalin (NGAL) reflects proximal and distal tubule injury and has been associated with CKD in several studies.^9–12^ Kidney injury molecule-1 (KIM-1), a marker of proximal tubule injury, has been associated with CKD in several cohorts and predicted future GFR decline in patients with HIV, CKD, or kidney transplant.^8,13–15^ Cell cycle arrest biomarkers, tissue inhibitor of metalloproteinase-2 (TIMP-2) and insulin-like growth factor-binding protein 7 (IGFBP-7), are urinary AKI biomarkers mainly studied in critically ill adults, with a few studies evaluating their utility in pediatric AKI.^16,17^ TIMP-2 has been associated with kidney function or CKD in children (CKiD).^18,19^ No studies have evaluated the usefulness of these biomarkers to predict later development of CKD or HTN in children treated for cancer. Early identification of children at high risk of CKD and HTN, ideally during or toward the end of cancer therapy, may help optimally determine kidney health follow-up and provide anticipatory guidance to providers and opportunities to prevent or treat long-term cisplatin nephrotoxicity.

We evaluated urine NGAL, KIM-1, TIMP-2, and IGFBP-7, collected during cisplatin infusions early in cancer therapy (early visit [EV]) and later in cancer therapy (late visit [LV]), for predicting signs of CKD and HTN at 3 months after cisplatin therapy completion. We chose this time point on the basis of the Kidney Disease Improving Global Outcomes (KDIGO) AKI guideline recommendation that AKI should be ascertained as either resolved or to have progressed to CKD by 3 months after an injurious AKI event.^20^ We also evaluated whether adding biomarkers to previously published clinical prediction models improves CKD and HTN prediction.

## Methods

### Study Design and Population

**Figure 1:**
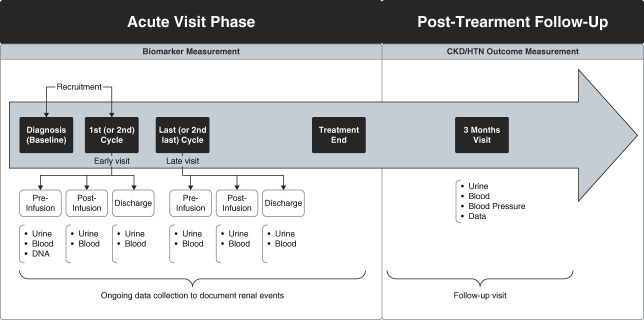
**Study time points from the ABLE study used for this analysis.** This diagram describes overall study flow from cancer diagnosis to the 3-year follow-up visit. Adapted from McMahon *et al.*, 2017.^21^ ABLE, Applying Biomarkers to Minimize Long-Term Effects of Childhood/Adolescent Cancer Treatment; HTN, hypertension. Figure was produced with Biorender.

This is a secondary analysis of data from the Applying Biomarkers to Minimize Long-Term Effects of Childhood/Adolescent Cancer Treatment (ABLE) Nephrotoxicity study, a 12-center, prospective, observational study of 159 children treated with cisplatin.^5,21^ Study methods have been published.^5,21^ The ABLE Nephrotoxicity study included patients younger than 18 years at cancer diagnosis and initiating cisplatin, with at most one prior cisplatin cycle (Figure 1). Participants were recruited either before the first *or s*econd cisplatin infusion cycle of cancer therapy. Individuals with a preexisting GFR <30 ml/min per 1.73 m^2^ or kidney transplantation were excluded. This analysis included patients with at least one biomarker measurement at EV (first *or* second cisplatin cycle) or LV (last *or* second-last cisplatin cycle) and at least one valid 3-month outcome measurement (CKD or HTN). Informed consent (and assent when appropriate) was obtained. The ABLE Nephrotoxicity study was approved by the research ethics board of each participating institution and adhered to the Declaration of Helsinki.

### Study Visits

The study included an acute study phase and a long-term follow-up phase (Figure 1). The acute phase involved data collection throughout cisplatin therapy and biospecimen collection during two cisplatin infusion cycles (EV or LV). At EV and LV, urine and blood were collected: pre-cisplatin infusion (preinfusion), postinfusion (<24 hours after infusion), and near hospital discharge (discharge). A follow-up visit was performed at 3 months after cisplatin therapy completion to ascertain CKD and HTN; every attempt was made to perform this visit when the patient was well (*i.e*., not admitted to hospital or clearly unwell). Participants' height, weight, and BP were measured three times (standardized methods; seated; size-appropriate cuffs; oscillometric device),^21^ expressed as percentiles.^22^ To minimize white coat effects, the two lowest systolic BP and corresponding diastolic BP measures were averaged.

### Laboratory Measurements and Biomarkers

SCr (isotope dilution mass spectrometry traceable assay) and urine albumin-to-creatinine ratio (uACR, mg/g) were measured at the Montreal Children's Hospital central biochemistry laboratory, Montreal, Canada. Urine biomarkers NGAL (ng/mg creatinine), KIM-1 (pg/mg creatinine), TIMP-2 (ng/ml), and IGFBP-7 (ng/ml) were measured at the Cincinnati Children's Hospital Medical Center Biomarker Laboratory, Ohio, with commercial ELISA kits (NGAL ELISA Kit 036, Bioporto, Grusbakken, Denmark; KIM-1, TIMP-2, IGFBP-7: Duoset DY1750. DY971, DY1334-05; R&D Systems, Inc., MN) using manufacturer's instructions.^23,24^ Inter and intra-assay coefficients of variation were 6.4% and 4.1% for NGAL, 10.7% and 6.1% for KIM-1, 8.6% and 5.4% for TIMP-2, and 9.9% and 4.6% for IGFBP-7, respectively. All measurements were performed blinded to clinical data. Biomarkers measured at preinfusion, postinfusion, and discharge time points at EV and LV were the main exposure variables evaluated to predict outcomes. NGAL and KIM-1 were indexed to urine creatinine concentrations, reported as ng/mg creatinine and pg/mg creatinine, respectively. TIMP-2 and IGFBP-7 were examined together in the form of their product (TIMP2×IGFBP-7, ng/ml^2^), as previously expressed.^25^ We also evaluated associations of sympercent change of EV and LV biomarkers (delta, from pre-cisplatin infusion to postinfusion and preinfusion to discharge time points) with outcomes. Sympercent change was the difference between the natural logs of the two numbers multiplied by 100.^26^

### Outcomes: 3-Month Post-Cisplatin CKD and HTN

CKD was defined on the basis of KDIGO guidelines: low eGFR (eGFR, fulfilling ≥stage 2 CKD) or elevated uACR.^20^ eGFR was calculated using the CKiD SCr-based equation.^27^ For participants older than 18–21 years, the average of the CKiD and adult CKD Epidemiology Collaboration equations was used.^27–29^ Low eGFR for age to qualify as stage 2 CKD was defined as eGFR<90 ml/min per 1.73 m^2^ for children ≥2 years old and defined using age-normative data with lower thresholds for children <2 years old.^27,30^ Albuminuria was defined as uACR ≥30 mg/g for children ≥2 years old and uACR ≥75 mg/g for children <2 years old, measured from a random sample.^20,31^

HTN was defined according to the 2017 American Academy of Pediatrics guidelines, using BP percentiles or BP thresholds, depending on participant age.^22,32^ Participants receiving antihypertensive medication were classified as hypertensive.

### Statistical Analyses

Prevalence of CKD and HTN at follow-up was calculated. Patient characteristics were compared between patients with and without outcomes using univariate analyses appropriate for variable distribution. Biomarker concentrations from the EV and LV time points and biomarker sympercent changes were compared in patients with and without outcomes, using the Mann–Whitney *U* test. The Skillings–Mack test was used to evaluate biomarker changes between time points. Biomarker diagnostic characteristics for predicting CKD and HTN were evaluated by calculating area under the receiver-operating characteristic curve (Area under the curve [AUC]) with associated 95% confidence intervals (CIs). Logistic regression with model AUC calculation was used to evaluate prediction of outcomes using biomarker combinations and the extent to which adding biomarkers to a clinical prediction model significantly increased AUC versus the clinical prediction model alone. The Delong test was used to evaluate the significance of adding biomarkers to clinical prediction models by evaluating AUC change.^33^ We performed secondary analyses to further evaluate the relationship of biomarkers with outcomes. We performed univariable and multivariable logistic regressions to evaluate unadjusted and adjusted associations between biomarkers expressed as ln-transformed continuous variables and biomarkers expressed as quartiles (with quartile 1 being the reference group) with CKD and HTN. In all models adjusting for clinical variables, we adjusted for previously determined clinical risk factors (previously published and described in results below).^34^ A *P* < 0.05 (two-tailed) was considered statistically significant. Stata version 15.1 (College Station, TX) was used for analyses.

## Results

### Participant Characteristics

**Figure 2:**
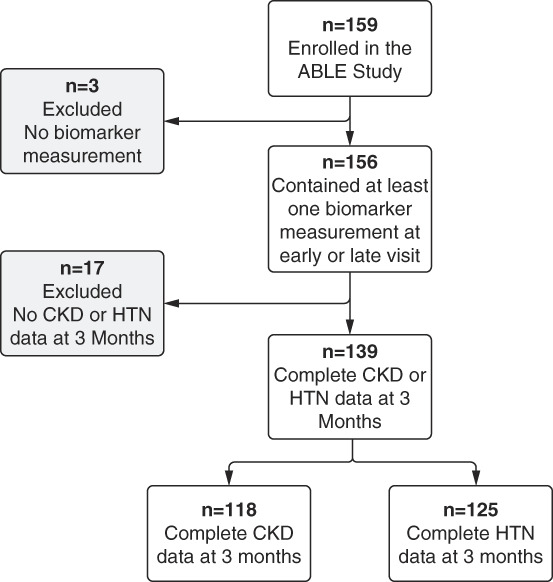
**Study flow chart.** The flow chart shows the total number of patients recruited into the ABLE Nephrotoxicity study and the cohort used for analysis. Figure was produced with Biorender.

Of 159 children enrolled in the cohort, 156 had biomarker measurements available at EV or LV (Figure 2). No patients with CKD at 3 months after therapy ended had CKD at baseline (pre-cisplatin therapy); no patients with HTN at 3 months had HTN had baseline. Three months after cancer therapy, 118 and 125 patients had CKD and HTN data, respectively (Figure 2). Median (interquartile range) age of the cohort at 3-month follow-up was 6 (3–12) years. At 3 months, 52 of 118 (44%) had CKD and 17 of 125 (14%) had HTN. Of 104 patients with 3-month data available for CKD and HTN, 9 (8.7%) had both CKD and HTN (representing 20.0% and 64.3% of patients with CKD and HTN, respectively). Of patients with CKD, 8 of52 (15.4%) and 43 of 52 (82.7%) fulfilled the low eGFR and high uACR criteria only, respectively; one patient fulfilled both criteria. All patients with low eGFR had eGFR >60 ml/min per 1.73 m^2^ (lowest eGFR was 61 ml/min per 1.73 m^2^). Four of the 44 patients with high uACR had macroalbuminuria (values of 99, 122, 388, and 1061 mg/mmol). Table 1 presents patient characteristics by 3-month CKD and HTN status; in general, children with outcomes (versus without) were younger and had a higher rate of kidney risk factors during cancer treatment.

**Table 1:** Characteristics of the cohort by CKD and hypertension status at the 3-month visit

Characteristic	3-mo Visit			
	CKD (*n*=52)	No CKD (*n*=66)	HTN (*n*=17)	No HTN (*n*=108)
**Baseline characteristics**				
Age at cisplatin treatment start, median (IQR), yr	3 (2–10)	7 (4–12)	2 (2–4)	7 (3–13)^k^
Age at cisplatin treatment start <3 yr, *No.* (%)	22 (42)	13 (20)^j^	10 (59)	25(23)^k^
Male, *No.* (%)	29 (56)	32 (48)	15 (88)	49(45)^k^
Race, *No.* (%)				
*Aboriginal*	2 (4)	2 (3)	0 (0.0)	7 (6)
*American Indian/Alaskan*	1 (2)	0 (0.0)	0 (0.0)	1 (0.9)
*White*	42 (81)	51 (77)	14 (82)	79 (73)
*Black*	1 (2)	1 (2)	1 (6)	3 (3)
*Asian*	2 (4)	8 (12)	1 (6)	10 (9)
*Mixed race*	4 (8)	4 (6)	1 (6)	7 (6)
*Hispanic*	0 (0.0)	0 (0.0)	0 (0.0)	1 (0.9)
Cancer type, *No.* (%)				
*Osteosarcoma*	8 (15)	18 (27)	1 (6)	29 (27)
*Germ cell tumor*	4 (8)	5 (8)	2 (12)	7 (6)
*Neuroblastoma*	17 (33)	20 (30)	6 (35)	23 (21)
*CNS tumor*^a^	20 (39)	18 (27)	6 (35)	40 (37)
*Hepatoblastoma*	3 (6)	3 (5)	2 (12)	7 (6)
*Other*^b^	0 (0.0)	2 (3)	0 (0.0)	2 (2)
Baseline GFR or eGFR, ml/min per 1.73 m^2^, mean (SD)^c^	155 (45)	141 (35)	148 (48)	138 (39)
Low baseline GFR or eGFR for age, *No.* (%)^d^	0 (0.0)	0 (0.0)	2 (12)	5 (5)
Kidney medical history, *No.* (%)^e^	6 (12)	5 (8)	0 (0.0)	5 (5)
Any nephrotoxic drug prior to first cisplatin, *No.* (%)^f^	12 (23)	8 (12)	2 (12)	19 (18)
*Vancomycin use before first cisplatin, No. (%)*	4 (8)	2 (3)	0 (0.0)	6 (6)
**During cisplatin/cancer treatment characteristics**				
Cancer involves one or both kidneys, *No.* (%)	7 (13)	2 (3)^j^	1 (6)	6 (6)
Flank (left or right), whole abdomen, or total body radiation given or planned, *No.* (%)	6 (12)	11 (17)	3 (18)	12 (11)
Total cumulative cisplatin dose, median (IQR), mg/m^2^	349 (254–410)	396 (294–446)	355 (240–410)	394 (283–466)
Stem cell transplant in chemotherapy protocol, *No.* (%)	29 (56)	24 (36)	12 (71)	35(32)^k^
Infection, *No.* (%)^g^	22 (42)	20 (30)	7 (41)	36 (33)
*Sepsis, No. (%)*	5 (10)	0 (0.0)^j^	0 (0.0)	5 (5)
*Kidney infection or UTI, No. (%)*^h^	4 (8)	2 (3)	3 (18)	4 (4)
PICU admission, *No.* (%)	4 (8)	5 (8)	4 (24)	5 (5)^j^
SCr-AKI during treatment, *No.* (%)	22 (42)	28 (42)	9 (53)	44 (41)
**Between last cisplatin infusion and follow-up visit characteristics**				
Nephrectomy, *No.* (%)	1 (2)	0 (0.0)	0 (0.0)	0 (0.0)
Nephrotoxic drug (in 1 mo prior to follow-up visit), *No.* (%)^i^	27 (52)	17(26)^k^	8 (47)	41 (38)
Acyclovir (in 1 mo prior to follow-up visit), *No.* (%)	7 (13)	7 (11)	2 (12)	11 (10)
Time between cisplatin treatment end and follow-up visit, median (IQR), d	88 (71–103)	85 (75–108)	85 (70–104)	91 (80–111)
BMI percentile at follow-up visit, median (IQR)	22 (5–58)	40 (19–61)	39 (20–52)	35 (8–63)

BMI, body mass index; CNS, central nervous system; HTN, hypertension; IQR, interquartile range; PICU, pediatric intensive care unit; SCr, serum creatinine; UTI, urinary tract infection.

a Central nervous system tumors: astrocytoma, choroid plexus tumor, ependymoma, medulloblastoma, primitive neuroectodermal tumor, and atypical teratoid/rhabdoid tumor.

b Other cancer: lymphoma and nasopharyngeal carcinoma.

c Baseline measured or eGFR was assessed using measured GFR if available or 24-hour creatinine clearance if unavailable; if both were unavailable, GFR was estimated (using the lowest 3-month pre-cisplatin serum creatinine level).

d Defined using age-based thresholds: aged ≤1 month, GFR <43 ml/min per 1.73 m^2^; aged 1–4 months, GFR <47 ml/min per 1.73 m^2^; aged 4–8 months, GFR <58 ml/min per 1.73 m^2^; aged 8 months-1 year, GFR <65 ml/min per 1.73 m^2^; aged 1–1.5 years, GFR <74 ml/min per 1.73 m^2^; aged 1.5–2 years, GFR <76 ml/min per 1.73 m^2^; and age >2 years, GFR <90 ml/min per 1.73 m^2^.

e Kidney medical history (on the basis of medical chart review): hypertension, treatment with antihypertensives, family history of kidney disease, CKD, dialysis, congenital kidney anomaly, kidney stones, vesicoureteral reflux, urinary tract infection, serum electrolyte abnormality requiring treatment or AKI.

f Nephrotoxic drugs include acyclovir, amphotericin, aminoglycosides (gentamycin, tobramycin, amikacin), vancomycin, angiotensin-converting enzyme inhibitor, ganciclovir/valganciclovir, ifosfamide, or methotrexate.

g Only infections with a positive culture and documentation were tabulated.

h Kidney infection or urinary tract infection includes any infection with a specimen originating from the kidneys or urinary tract infection.

i Nephrotoxic drugs include acyclovir, amphotericin, aminoglycosides (other than gentamycin, tobramycin and amikacin), vancomycin, angiotensin converting enzyme inhibitor, ganciclovir/valganciclovir, ifosfamide, or methotrexate.

j Stands for significant difference between Outcome and No Outcome groups: *P* < 0.05.

k Stands for significant difference between Outcome and No Outcome groups: *P* < 0.01.

### AKI Biomarker Associations with 3-Month CKD and HTN

Supplemental Table 1 shows biomarker concentrations in patients with and without CKD and HTN at 3 months. Overall, there were not many statistically significant differences between outcome groups (Supplemental Table 1). NGAL concentrations from EV discharge were approximately two-fold higher (*P* < 0.05) in patients with versus without 3-month CKD (Supplemental Table 1). EV preinfusion and discharge KIM-1 concentrations were 1.5 times (*P* < 0.05) and approximately four times higher (*P* < 0.05), respectively, in patients with versus without 3-month CKD (Supplemental Table 1). TIMP-2×IGFPB-7 concentrations at preinfusion of EV were approximately six times lower (*P* < 0.05) in patients with versus without 3-month HTN (Supplemental Table 1).

Table 2 presents the diagnostic characteristics of individual biomarkers and biomarker combinations from various study time points during cisplatin therapy to predict 3-month CKD and HTN. For CKD, biomarkers showed poor (<0.7) predictive values with AUCs only as high as 0.67 (Table 2). The most predictive biomarkers for 3-month CKD were NGAL and KIM-1 (combined) measured at discharge of the EV (AUC [95% CI]=0.67 [0.57 to 0.77]) and KIM-1 and TIMP-2×IGFBP-7 (combined) measured at preinfusion of the LV (AUC [95% CI]=0.66 [0.54 to 0.77] [Table 2]). Statistically significant AUCs (95% CIs above 0.5) for individual biomarkers to predict CKD were present for the NGAL EV discharge sample and KIM-1 EV preinfusion and discharge samples (Table 2).

**Table 2:** Area under the receiver-operating characteristic curve for AKI biomarkers measured during cisplatin therapy to predict CKD and hypertension at 3 months post-cisplatin

Biomarker and Time of Collection	AUC (95% CI) to Predict 3-mo CKD^a^^,^^b^	AUC (95% CI) to Predict 3-mo HTN^c^^,^^d^
**NGAL, ng/mg creatinine**		
EV preinfusion	0.55 (0.44 to 0.66)	0.47 (0.31 to 0.62)
EV postinfusion	0.58 (0.47 to 0.69)	0.48 (0.33 to 0.62)
EV discharge	0.65 (0.54 to 0.75)^e^	0.56 (0.36 to 0.75)
LV preinfusion	0.53 (0.40 to 0.65)	0.53 (0.36 to 0.70)
LV postinfusion	0.60 (0.48 to 0.72)	0.48 (0.29 to 0.66)
LV discharge	0.57 (0.44 to 0.69)	0.41 (0.23 to 0.59)
**KIM-1, pg/mg creatinine**		
EV preinfusion	0.62 (0.51 to 0.72)^e^	0.38 (0.22 to 0.54)
EV postinfusion	0.59 (0.48 to 0.69)	0.58 (0.43 to 0.73)
EV discharge	0.65 (0.55 to 0.76)^e^	0.53 (0.34 to 0.71)
LV preinfusion	0.57 (0.45 to 0.70)	0.49 (0.25 to 0.72)
LV postinfusion	0.61 (0.49 to 0.72)	0.64 (0.45 to 0.82)
LV discharge	0.42 (0.30 to 0.55)	0.59 (0.41 to 0.77)
**TIMP-2×IGFBP-7, (ng/ml)** ^**2**^ **/1000**		
EV preinfusion	0.48 (0.38 to 0.59)	0.70 (0.60 to 0.80)^e^
EV postinfusion	0.58 (0.47 to 0.69)	0.59 (0.43 to 0.74)
EV discharge	0.58 (0.48 to 0.69)	0.46 (0.29 to 0.62)
LV preinfusion	0.61 (0.49 to 0.72)	0.61 (0.44 to 0.78)
LV postinfusion	0.60 (0.48 to 0.72)	0.41 (0.25 to 0.57)
LV discharge	0.43 (0.31 to 0.55)	0.47 (0.33 to 0.61)
**NGAL (ng/mg creatinine) and KIM-1 (pg/mg creatinine)**		
EV preinfusion	0.62 (0.52 to 0.73)^e^	0.44 (0.29 to 0.60)
EV postinfusion	0.58 (0.47 to 0.69)	0.57 (0.41 to 0.72)
EV discharge	0.67 (0.57 to 0.77)^e^	0.57 (0.38 to 0.75)
LV preinfusion	0.52 (0.40 to 0.65)	0.58 (0.38 to 0.79)^f^
LV postinfusion	0.62 (0.50 to 0.74)	0.60 (0.40 to 0.80)
LV discharge	0.57 (0.45 to 0.70)^f^	0.58 (0.40 to 0.77)
**NGAL (ng/mg creatinine) and TIMP-2×IGFBP-7 (ng/ml)** ^**2**^ **/1000**		
EV preinfusion	0.59 (0.48 to 0.69)	0.71 (0.62 to 0.80)^f^
EV postinfusion	0.57 (0.46 to 0.68)	0.58 (0.42 to 0.74)
EV discharge	0.65 (0.54 to 0.76)^e^	0.53 (0.35 to 0.72)
LV preinfusion	0.63 (0.51 to 0.75)^e^	0.61 (0.43 to 0.78)
LV postinfusion	0.60 (0.48 to 0.72)	0.52 (0.33 to 0.72)
LV discharge	0.50 (0.38 to 0.63)	0.47 (0.33 to 0.60)
**KIM-1 (pg/mg creatinine) and TIMP-2×IGFBP-7 (ng/ml)** ^**2**^ **/1000**		
EV preinfusion	0.62 (0.52 to 0.73)^e^	0.70 (0.59 to 0.80)^f^
EV postinfusion	0.59 (0.48 to 0.70)	0.61 (0.46 to 0.76)
EV discharge	0.65 (0.54 to 0.75)^e^	0.50 (0.32 to 0.68)
LV preinfusion	0.66 (0.54 to 0.77)^e^	0.69 (0.55 to 0.84)^f^
LV postinfusion	0.61 (0.49 to 0.73)	0.65 (0.47 to 0.83)
LV discharge	0.46 (0.33 to 0.58)	0.66 (0.49 to 0.83)

Results stratified by time of cisplatin infusion. AUC, area under the curve; CI, confidence interval; EV, early visit; HTN, hypertension; IGFBP-7, insulin-like growth factor-binding protein 7; KIM-1, kidney injury molecule-1; LV, late visit; NGAL, neutrophil gelatinase-associated lipocalin; TIMP-2, tissue inhibitor of metalloproteinase-2.

a For CKD analyses, including neutrophil gelatinase-associated lipocalin or kidney injury molecule-1, 118 patients were included. Early visit preinfusion: *n*=113, early visit postinfusion: *n*=113, early visit discharge: *n*=109, late visit preinfusion: *n*=89, late visit postinfusion: *n*=92, late visit discharge: *n*=85.

b For CKD analyses, including tissue inhibitor of metalloproteinase-2×insulin-like growth factor-binding protein 7, 118 patients were included. Early visit preinfusion: *n*=117, early visit postinfusion: *n*=116, early visit discharge: *n*=111, late visit preinfusion: *n*=91, late visit postinfusion: *n*=93, late visit discharge: *n*=87.

c For hypertension analyses, including neutrophil gelatinase-associated lipocalin or kidney injury molecule-1, 125 patients were included. Early visit preinfusion: *n*=120, early visit postinfusion: *n*=121, early visit discharge: *n*=116, late visit preinfusion: *n*=98, late visit postinfusion: *n*=99, late visit discharge: *n*=95.

d For hypertension analyses, including tissue inhibitor of metalloproteinase-2×insulin-like growth factor-binding protein 7, 125 patients were included. Early visit preinfusion: *n*=124, early visit postinfusion: *n*=124, early visit discharge: *n*=118, late visit preinfusion: *n*=100, late visit postinfusion: *n*=100, late visit discharge: *n*=96.

e Area under the curves: 95% confidence interval did not cross 0.50.

f Statistically different than area under the curve of one biomarker alone as per the DeLong method.

For HTN prediction, AUCs were higher, but with values only as high as 0.71 (Table 2). The most predictive biomarker for 3-month HTN was NGAL combined with TIMP-2×IGFBP-7 at EV preinfusion (AUC [95% CI]: 0.71 [0.62 to 0.80]) (Table 2). Only EV preinfusion TIMP-2×IGFBP-7 sample had a statistically significant AUC to predict HTN (Table 2). Overall, combining biomarkers had at most a small effect to increase prediction of CKD and HTN compared with single-biomarker predictions (Table 2).

In secondary analyses of univariable and multivariable logistic regressions adjusting for previously identified clinical risk factors,^34^ individual biomarker associations (expressed as ln-transformed continuous variables or by quartiles concentration groups) were extremely similar in direction and statistical significance as presented in Table 2 AUC analyses (Supplemental Tables 2 and 3). In general, but not always, higher quartile concentrations of biomarkers had stronger associations with 3-month outcomes than lower biomarker quartile concentration groups (Supplemental Table 3).

### Associations of Sympercent Change in AKI Biomarkers with 3-Month CKD and HTN

Table 3 shows the sympercent change in biomarkers between patients with versus without CKD and with versus without HTN at 3 months. Sympercent change in NGAL was not significantly different between patients with versus without 3-month outcomes (Table 3). Sympercent change in KIM-1 from pre- to post-EV infusion was higher in participants with versus without HTN at 3 months post-cisplatin (increased pre- to post-EV by 58.22 pg/mg creatinine in patients with 3-month HTN; decreased by 28.28 pg/mg creatinine in patients without HTN; *P* < 0.05; Table 3). Sympercent change in KIM-1 from EV preinfusion to LV postinfusion was higher in participants with versus without 3-month HTN (Table 3). Sympercent changes in TIMP-2×IGFBP-7 from the LV pre- to postinfusion and from LV preinfusion to discharge were higher in patients with versus without 3-month CKD (Table 3). Sympercent change in TIMP-2×IGFBP-7 from EV preinfusion to discharge and from preinfusion of the EV to samples from the LV were higher in patients with versus without 3-month HTN (Table 3).

**Table 3:** Sympercent change in urine AKI biomarkers across study time points between patients with and without CKD and hypertension at 3 months post-cisplatin

Timepoint	3 mo	
	Median (IQR), *n*	Median (IQR), *n*
**NGAL, ng/mg creatinine**		
	CKD	No CKD
EV post—pre	8.18 (−61.75 to 70.01), 46	−12.23 (−68.91 to 46.41), 63
EV DC—pre	30.86 (−25.93 to 87.66), 44	7.95 (−48.38 to 66.92), 60
LV post—pre	21.38 (−132.64 to 99.03), 34	0.00 (−104.52 to 52.11), 51
LV DC—pre	27.31 (−95.05 to 85.80), 32	0.84 (−104.16 to 59.27), 46
LV post—EV pre	7.21 (−59.54 to 81.60), 37	−18.38 (−93.66 to 54.48), 51
LV DC—EV pre	13.65 (−86.18 to 86.50), 34	−11.09 (−122.89 to 95.64), 47
	HTN	No HTN
EV post—pre	25.15 (−37.96 to 46.48), 17	−12.25 (−68.08 to 50.10), 100
EV DC—pre	30.31 (−27.88 to 130.58), 15	24.87 (−48.38 to 76.86), 96
LV post—pre	8.63 (−92.97 to 99.03), 11	3.37 (−104.52 to 62.27), 83
LV DC—pre	48.92 (−116.41 to 145.33), 10	3.74 (−93.98 to 62.96), 80
LV post—EV pre	27.41 (−63.66 to 81.60), 13	−13.14 (−83.48 to 74.54), 82
LV DC—EV pre	38.59 (−7.13 to 90.83), 12	−15.71 (−121.87 to 79.19), 79
**KIM-1, pg/mg creatinine**		
	CKD	No CKD)
EV post—pre	−16.08 (−122.85 to 52.40), 46	−18.23 (−115.64 to 34.89), 63
EV DC—pre	104.68 (2.11–222.40), 44	82.83 (−1.86 to 183.53), 60
LV post—pre	12.44 (−56.43 to 69.65), 34	−28.17 (−74.65 to 44.89), 51
LV DC—pre	125.31 (24.81–170.53), 32	82.97 (−3.61 to 197.09), 46
LV post—EV pre	1.37 (−104.67 to 120.18), 37	−0.63 (−85.05 to 39.03), 51
LV DC—EV pre	105.82 (1.29–223.80), 34	119.30 (0.00–224.82), 47
	HTN	No HTN
EV post—pre	58.22 (4.00–115.57), 17	−28.28 (−127.00 to 21.52)^a^, 100
EV DC—pre	244.17 (−43.15 to 363.26), 15	101.67 (4.51–183.53), 96
LV post—pre	63.60 (−28.17 to 116.15), 11	−21.38 (−71.00 to 57.06), 83
LV DC—pre	146.80 (108.24–242.05), 10	101.25 (2.63–162.42), 80
LV post—EV pre	120.18 (−1.97 to 265.36), 13	−3.34 (−85.05 to 51.61)^a^, 82
LV DC—EV pre	170.02 (78.03–382.91), 12	108.26 (1.29–194.16), 79
**TIMP-2×IGFPB-7, (ng/ml)** ^2^ **/1000**		
	CKD	No CKD
EV post—pre	−158.67 (−334.57 to −28.15), 49	−216.91 (−379.92 to −27.75), 66
EV DC—pre	−23.79 (−184.16 to 140.34), 47	−43.78 (−326.85 to 178.77), 63
LV post—pre	−88.59 (−214.34 to 57.35), 37	−267.03 (−408.72 to −101.48)^a^, 51
LV DC—pre	48.66 (−210.32 to 193.02), 35	−173.15 (−327.23 to 58.77)^a^, 47
LV post—EV pre	−129.01 (−330.18 to 79.41), 40	−156.72 (−386.79 to −2.66), 52
LV DC—EV pre	−69.79 (−220.89 to 174.36), 38	−64.87 (−316.26 to 114.73), 48
	HTN	No HTN
EV post—pre	−84.27 (−356.05 to −2.18), 17	−210.39 (−370.18 to −44.69), 106
EV DC—pre	132.43 (−88.69 to 342.27), 16	−72.38 (−294.39 to 151.56)^a^, 101
LV post—pre	−47.37 (−183.49 to 121.16), 11	−195.54 (−351.76 to 43.02), 86
LV DC—pre	70.78 (−181.37 to 185.24), 10	−93.53 (−289.64 to 155.23), 83
LV post—EV pre	60.36 (−154.07 to 122.34), 13	−155.29 (−384.4 to −28.04)^a^, 86
LV DC—EV pre	65.97 (−128.06 to 243.54), 12	−104.74 (−281.63 to 110.96)^a^, 83

Results stratified by time of cisplatin infusion. DC, near hospital discharge; EV, early visit; HTN, hypertension; IGFBP-7, insulin-like growth factor-binding protein 7; IQR, interquartile range; KIM-1, kidney injury molecule-1; LV, late visit; NGAL, neutrophil gelatinase-associated lipocalin; Pre, pre-cisplatin infusion; Post, post-cisplatin infusion; TIMP-2, tissue inhibitor of metalloproteinase-2.

a Stands for significant difference between Outcome and No Outcome groups by Mann–Whitney *U* Test: *P* < 0.05.

Diagnostic characteristics of sympercent change in biomarkers were similar or slightly better for predicting 3-month CKD compared with biomarkers alone and were stronger predictors for HTN at 3 months (Supplemental Table 4). Sympercent change in KIM-1 from EV preinfusion to postinfusion predicted 3-month HTN with an AUC (95% CI) of 0.73 (0.60 to 0.87) (Supplemental Table 4). Sympercent change of TIMP-2×IGFBP-7 from EV preinfusion to LV postinfusion predicted 3-month HTN with an AUC (95% CI) of 0.72 (0.58 to 0.86) (Supplemental Table 4).

### Added Value of Biomarkers to Predict CKD and HTN

The previously published (our prior work)^34^ 3-month CKD clinical model included sex, age younger than 3 years at the start of cisplatin treatment, SCr-AKI during treatment, baseline GFR, and acyclovir use in the month preceding the 3-month visit (Table 4; AUC [95% CI], 0.68 [0.58 to 0.78]). Adding biomarkers to the CKD clinical model did not significantly improve AUCs (Table 4).

**Table 4:** Area under the receiver-operating characteristics curve of adding biomarkers to clinical models to predict CKD and hypertension at 3 months post-cisplatin treatment end

Time of Collection	AUC (95% CI) to Predict 3-mo CKD (*n*=118)	AUC (95% CI) to Predict 3-mo HTN (*n*=125)
Clinical model^a^	0.68 (0.58 to 0.78), 118	0.81 (0.72 to 0.91)
**Clinical model+NGAL, ng/mg creatinine**		
EV preinfusion	0.67 (0.57 to 0.77), 113	0.83 (0.74 to 0.92), 120
EV postinfusion	0.71 (0.62 to 0.81), 113	0.81 (0.71 to 0.90), 121
EV discharge	0.73 (0.64 to 0.83), 109	0.78 (0.68 to 0.89), 116
LV preinfusion	0.69 (0.59 to 0.80), 89	0.81 (0.70 to 0.92), 98
LV postinfusion	0.73 (0.62 to 0.83), 92	0.86 (0.75 to 0.97), 99
LV discharge	0.72 (0.61 to 0.82), 85	0.83 (0.70 to 0.95), 95
**Clinical model+KIM-1, pg/mg creatinine**		
EV preinfusion	0.70 (0.60 to 0.80), 113	0.80 (0.71 to 0.90), 120
EV postinfusion	0.69 (0.59 to 0.79), 113	0.82 (0.72 to 0.92), 121
EV discharge	0.74 (0.65 to 0.83), 109	0.78 (0.68 to 0.89), 116
LV preinfusion	0.70 (0.59 to 0.81), 89	0.81 (0.68 to 0.94), 98
LV postinfusion	0.73 (0.63 to 0.83), 92	0.83 (0.72 to 0.94), 99
LV discharge	0.72 (0.61 to 0.83), 85	0.82 (0.71 to 0.94), 95
**Clinical model+TIMP-2×IGFBP-7, (ng/ml)** ^**2**^ **/1000**		
EV preinfusion	0.67 (0.57 to 0.77), 117	0.88 (0.82 to 0.95)^b^, 124
EV postinfusion	0.69 (0.59 to 0.78), 116	0.82 (0.72 to 0.91), 124
EV discharge	0.70 (0.61 to 0.80), 111	0.79 (0.69 to 0.89), 118
LV preinfusion	0.73 (0.62 to 0.84), 91	0.79 (0.67 to 0.91), 100
LV postinfusion	0.73 (0.63 to 0.83), 93	0.83 (0.73 to 0.94), 100
LV discharge	0.73 (0.62 to 0.83), 87	0.86 (0.76 to 0.96), 96
**Clinical model+NGAL (ng/mg creatinine)+KIM-1, pg/mg creatinine**		
EV preinfusion	0.69 (0.59 to 0.79), 113	0.82 (0.73 to 0.92), 120
EV postinfusion	0.71 (0.61 to 0.81), 113	0.81 (0.71 to 0.91), 121
EV discharge	0.76 (0.68 to 0.85), 109	0.79 (0.68 to 0.89), 116
LV preinfusion	0.69 (0.58 to 0.80), 89	0.81 (0.68 to 0.94), 98
LV postinfusion	0.73 (0.63 to 0.84), 92	0.85 (0.73 to 0.96), 99
LV discharge	0.72 (0.61 to 0.83), 85	0.83 (0.71 to 0.96), 95
**Clinical model+NGAL (ng/mg creatinine)+TIMP-2×IGFBP-7, (ng/ml)** ^**2**^ **/1000**		
EV preinfusion	0.68 (0.58 to 0.78), 113	0.89 (0.83 to 0.95)^b^, 120
EV postinfusion	0.71 (0.62 to 0.81), 113	0.81 (0.72 to 0.91), 121
EV discharge	0.75 (0.66 to 0.84), 109	0.78 (0.67 to 0.88), 116
LV preinfusion	0.71 (0.60 to 0.82), 89	0.81 (0.70 to 0.92), 98
LV postinfusion	0.72 (0.62 to 0.83), 92	0.85 (0.74 to 0.97), 99
LV discharge	0.73 (0.62 to 0.84), 85	0.86 (0.76 to 0.96), 95
**Clinical model+KIM-1 (pg/mg creatinine)+TIMP-2×IGFBP-7, (ng/ml)** ^**2**^ **/1000**		
EV preinfusion	0.71 (0.61 to 0.81), 113	0.88 (0.81 to 0.94)^b^, 120
EV postinfusion	0.69 (0.59 to 0.79), 113	0.81 (0.71 to 0.91), 121
EV discharge	0.75 (0.66 to 0.84), 109	0.78 (0.68 to 0.89), 116
LV preinfusion	0.71 (0.60 to 0.82), 89	0.84 (0.73 to 0.96), 98
LV postinfusion	0.73 (0.62 to 0.83), 92	0.84 (0.74 to 0.94), 99
LV discharge	0.73 (0.62 to 0.84), 85	0.88 (0.79 to 0.96), 95

Results stratified by time of cisplatin infusion. For all area under the curves, 95% confidence interval did not cross 0.5. AUC, area under the curve; Discharge, near hospital discharge; EV, early visit; HTN, hypertension; IGFBP-7, insulin-like growth factor-binding protein 7; KIM-1, kidney injury molecule-1; LV, late visit; NGAL, neutrophil gelatinase-associated lipocalin; TIMP-2, tissue inhibitor of metalloproteinase-2.

a Variables included in the clinical model for CKD: sex, age at cisplatin treatment start <3 years, serum creatinine-AKI during treatment, baseline eGFR or measured GFR, and acyclovir use in 1 month before the 3-month visit; Variables included in the clinical model for hypertension: sex, age at cisplatin treatment start <3 years, and serum creatinine-AKI during cisplatin treatment.

b Statistically different from area under the curve of clinical model without biomarker included by the Delong method, *P* < 0.05.

For HTN, the clinical model contained sex, age younger than 3 years at the start of cisplatin treatment, and SCr-AKI during cisplatin treatment^34^ (AUC [95% CI], 0.81 [0.72 to 0.91]; Table 4). Adding TIMP-2×IGFBP-7 and adding NGAL with TIMP-2×IGFBP-7 combined from preinfusion of EV significantly increased the model AUC from 0.81 to 0.88 and 0.89, respectively (Table 4).

## Discussion

In this multicenter, prospective study, urine tubule injury biomarkers had a low-to-moderate ability to predict the development of CKD or HTN 3 months post-cisplatin. To our knowledge, this is the first study to evaluate associations of kidney injury biomarkers with post–cancer treatment kidney and BP abnormalities in pediatric cisplatin treatment.

There have been increasing numbers of publications on critically ill children and those treated with stem cell transplant showing that an AKI episode is associated with long-term CKD and HTN.^35,36^ Evidence of associations between AKI and long-term adverse kidney outcomes in children treated for solid tumors with various nephrotoxic chemotherapies is sporadic and unclear.^37^ This association is challenging to study; cancer treatment may last months to years and potentially include many AKI episodes. Defining time zero for studying kidney outcomes is challenging. Recent research has highlighted nephrotoxic drugs as major risk factors of poor long-term kidney outcomes in childhood cancer survivors.^38^ We focused on cisplatin, a well-known nephrotoxin with well-described tubular injury mechanisms and which remains necessary to use. We previously showed that kidney and BP abnormalities were very common 3 months after cisplatin therapy; however, AKI during therapy was only modestly associated. Cisplatin causes tubular damage. It is thus rational to evaluate tubular damage biomarkers during chemotherapy for enhancing prediction of later kidney outcomes. However, the ideal *timing* of *when* throughout the months of therapy to evaluate associations of biomarkers post-treatment outcomes was unknown. We sampled multiple time points, which are reproducible in research and/or clinic, surrounding cisplatin infusions early and late during cisplatin therapy. We acknowledged that biomarkers may be affected by many health events (*e.g*., infections; subacute injuries), by evaluating *acute biomarker change* (sympercent change) to reflect injury. Our biomarker choices to initially study were based on knowledge that they were commercially available and could undergo relatively rapid knowledge translation.

There has been little literature on our studied biomarkers in cancer settings. All three biomarkers are known to be produced by injured tubular cells and specifically by cisplatin damage in animals.^15,39–41^ In scant literature, we and others have evaluated associations of NGAL and KIM-1 with cisplatin-associated AKI in humans.^41–43^ Although there is knowledge on associations of AKI biomarkers with CKD, and less so with HTN, in non-cancer settings,^8–14,18,19,44–46^ there is little known on their prediction of later kidney outcomes in cancer and cisplatin populations. The time point of 3 months after cancer therapy is relevant; at that time, persistently abnormal kidney function may indicate CKD, and patients may be identified for specialty follow-up. In brief, we found that both NGAL and KIM-1, but not TIMP-2×IGFBP-7, measured at the first or second cisplatin cycle (EV), especially at the 3–5-day postinfusion discharge sample, were higher in patients who later had signs of CKD. The highest AUCs for CKD prediction were also from this time point, but were modest (<0.7). Moreover, none of the biomarkers added to 3-month CKD prediction, above the clinical model alone. Our findings on HTN differed; when preinfusion EV NGAL and TIMP-2×IGFBP-7 were added to the clinical model, 3-month HTN prediction increased significantly (by approximately 10%), suggesting there may be a role for biomarkers to identify patients at highest risk of later HTN, requiring close follow-up and nephroprotection. When biomarkers were expressed as sympercent change from preinfusion, the association with HTN was substantially stronger. When we designed this study, we believed that biomarkers measured later in cisplatin therapy might be more strongly associated with 3-month outcomes; we found the opposite. Perhaps later in cancer therapy, after repeated cisplatin and other nephrotoxins have been received, these biomarkers are less able to differentiate who will go on to have more permanent kidney damage because most patients have at least some subclinical tubular injuries. Earlier in therapy, biomarker elevation may indicate predisposition or fragility for later kidney or BP abnormalities. The stronger association with HTN was interesting. This finding may be due to high BP being a more reliable outcome than CKD because postural proteinuria and muscle mass variability may affect CKD measures.^47^ It would be beneficial to perform similar studies but using reference standard 24-hour ambulatory BP monitoring as the outcome. Post-AKI development of fibrosis from maladaptive cell repair may lead to increased renin and salt-sensitivity, which may be better captured by BP abnormalities, rather than CKD measures, which are affected by renal reserve.^48^ Future research should explore renin-angiotensin-aldosterone system abnormalities in the AKI to CKD pathogenesis after cisplatin.

In general, NGAL and KIM-1 at the early and late cisplatin visits increased after versus before infusion. However, TIMP-2×IGFBP7 in general *dropped* after infusion; lower concentrations *before* infusion and a larger drop in TIMP-2×IGFBP-7 from pre- to post-infusion (reflected by sympercent change) were associated with higher likelihood of 3-month HTN. These biomarkers have been associated with kidney function decline in children with CKD or kidney transplant.^18,19^ TIMP-2 and IGFBP-7 are cell cycle arrest proteins expressed in kidney tubular cells with injury; their upregulation may reflect their growth inhibitory functions because G_1_ cell cycle arrest is a consequence of AKI.^16^ The decrease in TIMP-2×IGFBP-7 observed in the acute injury setting in patients with 3-month kidney outcomes may be a protective mechanism to increase cell cycle activities in response to cell damage.^49^ These findings require confirmation because they suggest a different way of interpreting TIMP-2×IGFBP-7 compared with use in the AKI diagnosis setting.

Our study begins to address the lack of AKI biomarker studies in the pediatric cancer population and the goal to promote early detection and treatment of kidney disease in pediatric cancer survivors. Study strengths included the prospective, multicenter design; the use of multiple evaluation time points; rigorous evaluation of clinically relevant kidney outcomes; and our building upon previous work on 3-month outcome clinical prediction. Although this is one of the largest cisplatin-specific pediatric cohorts published, the sample size limited the ability to adjust for additional covariates and to calculate precise association estimates. While our study has provided valuable insights into the association of urine biomarkers with CKD, it is pertinent to note that we observed predominantly mild forms of CKD within our cohort. We acknowledge that the associations might have been more pronounced if the cohort had included a higher number of participants with severe CKD. Another limitation is that the albumin-to-creatinine ratio was measured from a single spot urine sample; although this is supported by guidelines, children are known to have postural proteinuria.^20,50^ In a multicenter setting of childhood cancer treatment, performing repeated first morning urine samples was felt to be infeasible. We acknowledge that examining outcomes only 3 months after cisplatin therapy may not capture the full spectrum of CKD progression. Although the KDIGO guidelines recommend evaluating for CKD at approximately 3 months after AKI events, if the kidney and BP abnormalities we found are not yet CKD (but rather, acute changes or still recovering from AKI), this may have affected the relationship between the biomarkers we measured and our 3-month outcomes. We did, however, greatly attempt to perform 3-month visits when patients were well to try to avoid this problem. Our secondary analyses using adjusted logistic regression for association of biomarker quartiles with 3-month outcomes revealed that the higher quartile concentration groups were sometimes, but not always, more strongly associated with the outcomes. Unfortunately, the sample size limited the ability to evaluate this with confidence. Future studies should attempt to confirm these findings and potentially attempt to include participants with more severe cisplatin-associated kidney injury who would be more likely to have higher biomarker concentrations. Most participants were White, limiting generalizability of our findings, which we hope studies in other geographical regions will be able to address.

Individually measured urine NGAL, KIM-1, and TIMP-2×IGFBP-7 had low to modest predictive ability for 3-month signs of CKD and HTN; however, adding them to a clinical prediction model for 3-month HTN enhanced prediction. When feasible, future clinical prediction models could include urine tubule injury markers to validate these findings and identify patients requiring targeted early intervention. Other biomarkers that may more precisely identify early chronic injury should be studied, including metabolomics and possibly biomarkers evaluating fibrosis.

## Supplementary Material

## Data Availability

All data are included in the manuscript and/or supporting information.

## Supplemental Material

Supplemental Table 1. Urine AKI biomarker concentrations during cisplatin therapy in participants with or without CKD or HTN at 3 months post-cisplatin treatment end.

Supplemental Table 2. Univariable and multivariable associations of biomarkers with CKD and HTN at 3 months post-cisplatin treatment end.

Supplemental Table 3. Univariable and multivariable associations of biomarker quartiles with CKD and HTN at 3 months post-cisplatin treatment end.

Supplemental Table 4. Area under the receiver-operating characteristics curve for sympercent change in urine AKI biomarkers to predict CKD and HTN at 3 months post-cisplatin treatment.
