# Supplementary material for: Tubular Injury Biomarkers to Predict CKD and Hypertension at 3 Months Post-Cisplatin in Children
Source: Kidney360. 2024 Apr 26;5(6):821–33. doi: 10.34067/KID.0000000000000448 (PMC11219117; doi:10.34067/KID.0000000000000448)
Supplement: Supplementary file 1 [file kidney360-5-821-s001.pdf]

ASN American Society of Nephrology
JASN Journal of the American Society of Nephrology
KIDNEY360 Accessing Our World From Every Angle
CJASN Clinical Journal of the American Society of Nephrology

ASN Journal Disclosure Form

As per ASN journal policy, I have disclosed any financial relationship or commitment held by myself and/or my spouse/partner in the past 36 months as included below. I have listed my Current Employer below to indicate there is a relationship requiring disclosure. If no relationship exists, my Current Employer is not listed.

T. Blydt-Hansen reports the following:
Employer: BC Children's Hospital; BC Transplant; University of British Columbia; Ownership Interest: Hydrogenics; Apple ; Royal Bank of Canada; Research Funding: Canadian Institutes of Health Research; Astellas Canada; Child and Family Research Institute; Children's Hospital of Manitoba Research Institute; Transplant Research Foundation of BC, Eurofins, Kidney Foundation of Canada, National Institutes of Health.; and Advisory or Leadership Role: Canadian National Transplant Research Program; North American Pediatric Transplant and Collaborative Studies; BC Transplant;; International Pediatric Transplant Association, Council member.

I understand that the information above will be published within the journal article, if accepted, and that failure to comply and/or to accurately and completely report the potential financial conflicts of interest could lead to the following: 1) Prior to publication, article rejection, or 2) Post-publication, sanctions ranging from, but not limited to, issuing a correction, reporting the inaccurate information to the authors' institution, banning authors from submitting work to ASN journals for varying lengths of time, and/or retraction of the published work.

Name: Tom D. Blydt-Hansen
Manuscript ID: K360-2023-000729R1
Manuscript Title: Tubular Injury Biomarkers to Predict Chronic Kidney Disease and Hypertension at 3-Months Post-Cisplatin in Children
Date of Completion: April 4, 2024
Disclosure Updated Date: March 27, 2024

ASN American Society of Nephrology
JASN Journal of the American Society of Nephrology
KIDNEY360 Accessing Our World From Every Angle
CJASN Clinical Journal of the American Society of Nephrology

ASN Journal Disclosure Form

As per ASN journal policy, I have disclosed any financial relationship or commitment held by myself and/or my spouse/partner in the past 36 months as included below. I have listed my Current Employer below to indicate there is a relationship requiring disclosure. If no relationship exists, my Current Employer is not listed.

B. Carleton reports the following:
Employer: University of British Columbia; Consultancy: Dynacare Specialized Diagnostics; Research Funding: Only research grants from federal and provincial agencies except for a peer-reviewed federal grant with an industry partner (Dynacare Specialized Diagnostic) which ended in June 2021.; Advisory or Leadership Role: Advisor, Neopharm Labs; Advisor, Dynacare Specialized Diagnostics; and Other Interests or Relationships: Chair, Board of Directors, Rare Disease Foundation (uncompensated, volunteer position).

I understand that the information above will be published within the journal article, if accepted, and that failure to comply and/or to accurately and completely report the potential financial conflicts of interest could lead to the following: 1) Prior to publication, article rejection, or 2) Post-publication, sanctions ranging from, but not limited to, issuing a correction, reporting the inaccurate information to the authors’ institution, banning authors from submitting work to ASN journals for varying lengths of time, and/or retraction of the published work.

Name: Bruce Carleton
Manuscript ID: K360-2023-000729R1
Manuscript Title: Tubular Injury Biomarkers to Predict Chronic Kidney Disease and Hypertension at 3-Months Post-Cisplatin in Children
Date of Completion: February 15, 2024
Disclosure Updated Date: February 15, 2024

ASN Journal Disclosure Form

As per ASN journal policy, I have disclosed any financial relationship or commitment held by myself and/or my spouse/partner in the past 36 months as included below. I have listed my Current Employer below to indicate there is a relationship requiring disclosure. If no relationship exists, my Current Employer is not listed.

H. Chui has nothing to disclose.

I understand that the information above will be published within the journal article, if accepted, and that failure to comply and/or to accurately and completely report the potential financial conflicts of interest could lead to the following: 1) Prior to publication, article rejection, or 2) Post-publication, sanctions ranging from, but not limited to, issuing a correction, reporting the inaccurate information to the authors' institution, banning authors from submitting work to ASN journals for varying lengths of time, and/or retraction of the published work.

Name: Hayton Chui
Manuscript ID: K360-2023-000729R1
Manuscript Title: Tubular Injury Biomarkers to Predict Chronic Kidney Disease and Hypertension at 3-Months Post-Cisplatin in Children
Date of Completion: April 10, 2024
Disclosure Updated Date: April 10, 2024

ASN American Society of Nephrology
JASN Journal of the American Society of Nephrology
KIDNEY360 Accessing Our World From Every Angle
CJASN Clinical Journal of the American Society of Nephrology

ASN Journal Disclosure Form

As per ASN journal policy, I have disclosed any financial relationship or commitment held by myself and/or my spouse/partner in the past 36 months as included below. I have listed my Current Employer below to indicate there is a relationship requiring disclosure. If no relationship exists, my Current Employer is not listed.

V. Cockovski reports the following:
Employer: Hospital for Sick Children

I understand that the information above will be published within the journal article, if accepted, and that failure to comply and/or to accurately and completely report the potential financial conflicts of interest could lead to the following: 1) Prior to publication, article rejection, or 2) Post-publication, sanctions ranging from, but not limited to, issuing a correction, reporting the inaccurate information to the authors' institution, banning authors from submitting work to ASN journals for varying lengths of time, and/or retraction of the published work.

Name: Vedran Cockovski
Manuscript ID: K360-2023-000729R1
Manuscript Title: Tubular Injury Biomarkers to Predict Chronic Kidney Disease and Hypertension at 3-Months Post-Cisplatin in Children
Date of Completion: April 3, 2024
Disclosure Updated Date: May 23, 2023

ASN American Society of Nephrology JASN Journal of the American Society of Nephrology KIDNEY360 Accessing Our World From Every Angle CJASN Clinical Journal of the American Society of Nephrology

ASN Journal Disclosure Form

As per ASN journal policy, I have disclosed any financial relationship or commitment held by myself and/or my spouse/partner in the past 36 months as included below. I have listed my Current Employer below to indicate there is a relationship requiring disclosure. If no relationship exists, my Current Employer is not listed.

G. Cuvelier reports the following:
Employer: Alberta Children's Hospital / Alberta Health Services

I understand that the information above will be published within the journal article, if accepted, and that failure to comply and/or to accurately and completely report the potential financial conflicts of interest could lead to the following: 1) Prior to publication, article rejection, or 2) Post-publication, sanctions ranging from, but not limited to, issuing a correction, reporting the inaccurate information to the authors' institution, banning authors from submitting work to ASN journals for varying lengths of time, and/or retraction of the published work.

Name: Geoffrey D.e. Cuvelier
Manuscript ID: K360-2023-000729R1
Manuscript Title: Tubular Injury Biomarkers to Predict Chronic Kidney Disease and Hypertension at 3-Months Post-Cisplatin in Children
Date of Completion: April 3, 2024
Disclosure Updated Date: April 3, 2024

ASN
American Society of Nephrology
JASN
Journal of the American Society of Nephrology
KIDNEY360
Accessing Our World From Every Angle
CJASN
Clinical Journal of the American Society of Nephrology

ASN Journal Disclosure Form

As per ASN journal policy, I have disclosed any financial relationship or commitment held by myself and/or my spouse/partner in the past 36 months as included below. I have listed my Current Employer below to indicate there is a relationship requiring disclosure. If no relationship exists, my Current Employer is not listed.

R. Huang has nothing to disclose.

I understand that the information above will be published within the journal article, if accepted, and that failure to comply and/or to accurately and completely report the potential financial conflicts of interest could lead to the following: 1) Prior to publication, article rejection, or 2) Post-publication, sanctions ranging from, but not limited to, issuing a correction, reporting the inaccurate information to the authors' institution, banning authors from submitting work to ASN journals for varying lengths of time, and/or retraction of the published work.

Name: Ryan S Huang
Manuscript ID: K360-2023-000729R1
Manuscript Title: Tubular Injury Biomarkers to Predict Chronic Kidney Disease and Hypertension at 3-Months Post-Cisplatin in Children
Date of Completion: February 14, 2024
Disclosure Updated Date: February 14, 2024

ASN
American Society of Nephrology
JASN
Journal of the American Society of Nephrology
KIDNEY360
Accessing Our World From Every Angle
CJASN
Clinical Journal of the American Society of Nephrology

ASN Journal Disclosure Form

As per ASN journal policy, I have disclosed any financial relationship or commitment held by myself and/or my spouse/partner in the past 36 months as included below. I have listed my Current Employer below to indicate there is a relationship requiring disclosure. If no relationship exists, my Current Employer is not listed.

A. Lebel has nothing to disclose.

I understand that the information above will be published within the journal article, if accepted, and that failure to comply and/or to accurately and completely report the potential financial conflicts of interest could lead to the following: 1) Prior to publication, article rejection, or 2) Post-publication, sanctions ranging from, but not limited to, issuing a correction, reporting the inaccurate information to the authors' institution, banning authors from submitting work to ASN journals for varying lengths of time, and/or retraction of the published work.

Name: Asaf Lebel Asaf Lebel
Manuscript ID: K360-2023-000729R1
Manuscript Title: Tubular Injury Biomarkers to Predict Chronic Kidney Disease and Hypertension at 3-Months Post-Cisplatin in Children
Date of Completion: February 15, 2024
Disclosure Updated Date: February 15, 2024

ASN Journal Disclosure Form

As per ASN journal policy, I have disclosed any financial relationship or commitment held by myself and/or my spouse/partner in the past 36 months as included below. I have listed my Current Employer below to indicate there is a relationship requiring disclosure. If no relationship exists, my Current Employer is not listed.

J. Lee has nothing to disclose.

I understand that the information above will be published within the journal article, if accepted, and that failure to comply and/or to accurately and completely report the potential financial conflicts of interest could lead to the following: 1) Prior to publication, article rejection, or 2) Post-publication, sanctions ranging from, but not limited to, issuing a correction, reporting the inaccurate information to the authors' institution, banning authors from submitting work to ASN journals for varying lengths of time, and/or retraction of the published work.

Name: Jasmine Lee
Manuscript ID: K360-2023-000729R1
Manuscript Title: Tubular Injury Biomarkers to Predict Chronic Kidney Disease and Hypertension at 3-Months Post-Cisplatin in Children
Date of Completion: April 4, 2024
Disclosure Updated Date: April 4, 2024

ASN American Society of Nephrology
JASN Journal of the American Society of Nephrology
KIDNEY360 Accessing Our World From Every Angle
CJASN Clinical Journal of the American Society of Nephrology

ASN Journal Disclosure Form

As per ASN journal policy, I have disclosed any financial relationship or commitment held by myself and/or my spouse/partner in the past 36 months as included below. I have listed my Current Employer below to indicate there is a relationship requiring disclosure. If no relationship exists, my Current Employer is not listed.

C. Mammen has nothing to disclose.

I understand that the information above will be published within the journal article, if accepted, and that failure to comply and/or to accurately and completely report the potential financial conflicts of interest could lead to the following: 1) Prior to publication, article rejection, or 2) Post-publication, sanctions ranging from, but not limited to, issuing a correction, reporting the inaccurate information to the authors' institution, banning authors from submitting work to ASN journals for varying lengths of time, and/or retraction of the published work.

Name: Cherry Mammen
Manuscript ID: K360-2023-000729R1
Manuscript Title: Tubular Injury Biomarkers to Predict Chronic Kidney Disease and Hypertension at 3-Months Post-Cisplatin in Children
Date of Completion: April 3, 2024
Disclosure Updated Date: May 22, 2023

ASN American Society of Nephrology | JASN Journal of the American Society of Nephrology | KIDNEY360 Accessing Our World From Every Angle | CJASN Clinical Journal of the American Society of Nephrology

ASN Journal Disclosure Form

As per ASN journal policy, I have disclosed any financial relationship or commitment held by myself and/or my spouse/partner in the past 36 months as included below. I have listed my Current Employer below to indicate there is a relationship requiring disclosure. If no relationship exists, my Current Employer is not listed.

K. McMahon reports the following:
Employer: Research Institute of the McGill University Health Centre; Innovaderm Research Inc.

I understand that the information above will be published within the journal article, if accepted, and that failure to comply and/or to accurately and completely report the potential financial conflicts of interest could lead to the following: 1) Prior to publication, article rejection, or 2) Post-publication, sanctions ranging from, but not limited to, issuing a correction, reporting the inaccurate information to the authors' institution, banning authors from submitting work to ASN journals for varying lengths of time, and/or retraction of the published work.

Name: Kelly McMahon
Manuscript ID: K360-2023-000729R1
Manuscript Title: Tubular Injury Biomarkers to Predict Chronic Kidney Disease and Hypertension at 3-Months Post-Cisplatin in Children
Date of Completion: March 4, 2024
Disclosure Updated Date: March 4, 2024

RL-24 (2023-10)

RELEVÉ 24
Frais de garde d'enfants

Année	Code du relevé	Nº du dernier relevé transmis	
2023	R		952 926 030

Nom de chacun des enfants – Nom de famille	Prénom	Date de naissance A (AAAA MM JJ)	Nombre de jours de garde B.1	Nombre de semaines de pensionnat ou de colonie de vacances B.2	Total des frais payés C	Frais ne donnant pas droit au crédit d'impôt D	Frais donnant droit au crédit d'impôt E
Isabal	Victor Ivan	2018 01 26	170	0	7928 35	9 35	7919 00

Instructions et explications relatives aux cases du relevé 24

Pour connaître les conditions donnant droit au crédit d'impôt pour frais de garde d'enfants, consultez le guide de la déclaration à la ligne 455.

S'il y a lieu, reportez les montants inscrits aux cases de ce relevé aux lignes correspondantes de votre déclaration de revenus.

Nom de chacun des enfants (lignes 26.3 à 37.3 de l'annexe C)

A Date de naissance de l'enfant (lignes 26 à 37 de l'annexe C)

B.1 Nombre de jours de garde pour lesquels vous avez versé une somme à titre de frais de garde pour l'enfant

B.2 Nombre de semaines où l'enfant a fréquenté un pensionnat ou une colonie de vacances

C Total des frais payés. Ce montant représente le total des montants inscrits aux cases D et E.

D Frais **ne donnant pas droit** au crédit d'impôt pour frais de garde d'enfants, y compris la contribution réduite fixée par le gouvernement.

Si vous avez payé la contribution réduite durant l'année et que la case D est vide ou que le montant qui y est inscrit est inférieur au montant de la contribution réduite que vous avez payée pour l'enfant, nous vous invitons à communiquer avec votre fournisseur de services de garde. Il pourrait devoir vous transmettre un relevé 24 modifié.

E Frais **donnant droit** au crédit d'impôt pour frais de garde d'enfants (lignes 26.1 à 37.1 de l'annexe C)

G Numéro d'assurance sociale de la personne (généralement le parent) qui a payé les frais de garde

H Numéro d'identification du fournisseur des services de garde (lignes 26.2 à 37.2 de l'annexe C)

Important

Toute personne qui offre ou fournit des services de garde contre rémunération relativement à un enfant qui n'est pas admis à l'école ni assujetti à l'obligation de l'être, en contrepartie d'une contribution du parent, doit obtenir un permis du ministère de la Famille ou une reconnaissance d'un bureau coordonnateur de la garde en milieu familial. Certaines exceptions prévues à la Loi sur les services de garde éducatifs à l'enfance peuvent s'appliquer.

Une personne qui exploite un service de garde et qui ne détient pas de permis ni de reconnaissance alors qu'elle devait le faire exploite son service de garde illégalement.

PARENT	SERVICES DE GARDE
G- Numéro d'assurance sociale de la personne qui a payé les frais de garde	H- Numéro d'identification du fournisseur des services de garde
273 992 537	2241123464

Nom et adresse du fournisseur des services de garde

Garderikiri
100 rue Kepler suite 6
Châteauguay
QC J6K 5E4

Nom de famille, prénom et adresse de la personne qui a payé les frais de garde

Palijan, Ana
350 Doucet
Chateauguay
QC J6J 1P1

FS 2324004

REVENU QUÉBEC

2 – Copie du particulier qui a payé les frais de garde (Vous devez inclure ces données à votre déclaration de revenus et conserver cette copie.)

150V ZZ 49534886

Relevé officiel – Revenu Québec
Formulaire prescrit

ASN American Society of Nephrology | JASN Journal of the American Society of Nephrology | KIDNEY360 Accessing Our World From Every Angle | CJASN Clinical Journal of the American Society of Nephrology

ASN Journal Disclosure Form

As per ASN journal policy, I have disclosed any financial relationship or commitment held by myself and/or my spouse/partner in the past 36 months as included below. I have listed my Current Employer below to indicate there is a relationship requiring disclosure. If no relationship exists, my Current Employer is not listed.

M. Pinsk reports the following:
Employer: Shared Health Manitoba and University of Manitoba; Consultancy: CADTH (Canadian Agency for Drugs and Technologies in Health) - consultant; Expert witness for legal consultations (multiple);; Research Funding: Apellis; Advisory or Leadership Role: University Medical Group (U Manitoba) Board Member; and Other Interests or Relationships: Canadian Society of Nephrology; Canadian Association of Pediatric Nephrology.

I understand that the information above will be published within the journal article, if accepted, and that failure to comply and/or to accurately and completely report the potential financial conflicts of interest could lead to the following: 1) Prior to publication, article rejection, or 2) Post-publication, sanctions ranging from, but not limited to, issuing a correction, reporting the inaccurate information to the authors' institution, banning authors from submitting work to ASN journals for varying lengths of time, and/or retraction of the published work.

Name: Maury N. Pinsk
Manuscript ID: K360-2023-000729R1,
Manuscript Title: Tubular Injury Biomarkers to Predict Chronic Kidney Disease and Hypertension at 3-Months Post-Cisplatin in Children
Date of Completion: February 15, 2024
Disclosure Updated Date: February 15, 2024

ASN American Society of Nephrology
JASN Journal of the American Society of Nephrology
KIDNEY360 Accessing Our World From Every Angle
CJASN Clinical Journal of the American Society of Nephrology

ASN Journal Disclosure Form

As per ASN journal policy, I have disclosed any financial relationship or commitment held by myself and/or my spouse/partner in the past 36 months as included below. I have listed my Current Employer below to indicate there is a relationship requiring disclosure. If no relationship exists, my Current Employer is not listed.

S. Rassekh reports the following:
Employer: British Columbia's Children's Hospital (Provincial Health Services Association).; University of British Columbia; Consultancy: Knowles Intellectual Property Strategies (KIPS LLC); and Honoraria: Jazz Pharmaceuticals.

I understand that the information above will be published within the journal article, if accepted, and that failure to comply and/or to accurately and completely report the potential financial conflicts of interest could lead to the following: 1) Prior to publication, article rejection, or 2) Post-publication, sanctions ranging from, but not limited to, issuing a correction, reporting the inaccurate information to the authors' institution, banning authors from submitting work to ASN journals for varying lengths of time, and/or retraction of the published work.

Name: Shahrad Rod Rassekh
Manuscript ID: K360-2023-000729R1
Manuscript Title: Tubular Injury Biomarkers to Predict Chronic Kidney Disease and Hypertension at 3-Months Post-Cisplatin in Children
Date of Completion: February 16, 2024
Disclosure Updated Date: February 16, 2024

ASN American Society of Nephrology | JASN Journal of the American Society of Nephrology | KIDNEY360 Accessing Our World From Every Angle | CJASN Clinical Journal of the American Society of Nephrology

ASN Journal Disclosure Form

As per ASN journal policy, I have disclosed any financial relationship or commitment held by myself and/or my spouse/partner in the past 36 months as included below. I have listed my Current Employer below to indicate there is a relationship requiring disclosure. If no relationship exists, my Current Employer is not listed.

C. Ross reports the following:
Employer: University of British Columbia

I understand that the information above will be published within the journal article, if accepted, and that failure to comply and/or to accurately and completely report the potential financial conflicts of interest could lead to the following: 1) Prior to publication, article rejection, or 2) Post-publication, sanctions ranging from, but not limited to, issuing a correction, reporting the inaccurate information to the authors' institution, banning authors from submitting work to ASN journals for varying lengths of time, and/or retraction of the published work.

Name: Colin J. Ross
Manuscript ID: K360-2023-000729R1
Manuscript Title: Tubular Injury Biomarkers to Predict Chronic Kidney Disease and Hypertension at 3-Months Post-Cisplatin in Children
Date of Completion: April 3, 2024
Disclosure Updated Date: April 3, 2024

ASN
American Society of Nephrology
JASN
Journal of the American Society of Nephrology
KIDNEY360
Accessing Our World From Every Angle
CJASN
Clinical Journal of the American Society of Nephrology

ASN Journal Disclosure Form

As per ASN journal policy, I have disclosed any financial relationship or commitment held by myself and/or my spouse/partner in the past 36 months as included below. I have listed my Current Employer below to indicate there is a relationship requiring disclosure. If no relationship exists, my Current Employer is not listed.

K. Schultz reports the following:
Employer: University of British Columbia; Consultancy: BMS; Jazz; AlloVIR; Seres; Honoraria: Jazz; BMS; Sanofi; Advisory or Leadership Role: Advisory of Sanofi, Jazz, Novartis; and Other Interests or Relationships: DSMB BMS/Juno; DSMB AlloVIR; DSMB Seres.

I understand that the information above will be published within the journal article, if accepted, and that failure to comply and/or to accurately and completely report the potential financial conflicts of interest could lead to the following: 1) Prior to publication, article rejection, or 2) Post-publication, sanctions ranging from, but not limited to, issuing a correction, reporting the inaccurate information to the authors' institution, banning authors from submitting work to ASN journals for varying lengths of time, and/or retraction of the published work.

Name: Kirk Raymond Schultz
Manuscript ID: K360-2023-000729R1
Manuscript Title: "Tubular Injury Biomarkers to Predict Chronic Kidney Disease and Hypertension at 3-Months Post-Cisplatin in Children,"
Date of Completion: April 4, 2024
Disclosure Updated Date: February 15, 2024

ASN American Society of Nephrology
JASN Journal of the American Society of Nephrology
KIDNEY360 Accessing Our World From Every Angle
CJASN Clinical Journal of the American Society of Nephrology

ASN Journal Disclosure Form

As per ASN journal policy, I have disclosed any financial relationship or commitment held by myself and/or my spouse/partner in the past 36 months as included below. I have listed my Current Employer below to indicate there is a relationship requiring disclosure. If no relationship exists, my Current Employer is not listed.

R. Tsuyuki reports the following:
Employer: University of Alberta; Consultancy: Shoppers Drug Mart; Emergent Biosolutions; Research Funding: Sanofi Genzyme Canada, Merck Canada; Honoraria: Merck Canada Inc; Emergent Biosolutions, Novo Nordisk; and Advisory or Leadership Role: Editor-in-Chief, Canadian Pharmacists Journal;; President and Chair, Board of Directors, Hypertension Canada.

I understand that the information above will be published within the journal article, if accepted, and that failure to comply and/or to accurately and completely report the potential financial conflicts of interest could lead to the following: 1) Prior to publication, article rejection, or 2) Post-publication, sanctions ranging from, but not limited to, issuing a correction, reporting the inaccurate information to the authors' institution, banning authors from submitting work to ASN journals for varying lengths of time, and/or retraction of the published work.

Name: Ross T. Tsuyuki
Manuscript ID: K360-2023-000729R1
Manuscript Title: Tubular Injury Biomarkers to Predict Chronic Kidney Disease and Hypertension at 3-Months Post-Cisplatin in Children
Date of Completion: February 15, 2024
Disclosure Updated Date: February 15, 2024

ASN American Society of Nephrology
JASN Journal of the American Society of Nephrology
KIDNEY360 Accessing Our World From Every Angle
CJASN Clinical Journal of the American Society of Nephrology

ASN Journal Disclosure Form

As per ASN journal policy, I have disclosed any financial relationship or commitment held by myself and/or my spouse/partner in the past 36 months as included below. I have listed my Current Employer below to indicate there is a relationship requiring disclosure. If no relationship exists, my Current Employer is not listed.

S. Wang reports the following:
Employer: The Hospital for Sick Children

I understand that the information above will be published within the journal article, if accepted, and that failure to comply and/or to accurately and completely report the potential financial conflicts of interest could lead to the following: 1) Prior to publication, article rejection, or 2) Post-publication, sanctions ranging from, but not limited to, issuing a correction, reporting the inaccurate information to the authors' institution, banning authors from submitting work to ASN journals for varying lengths of time, and/or retraction of the published work.

Name: Stella Qiongbin Wang
Manuscript ID: K360-2023-000729R1
Manuscript Title: Tubular Injury Biomarkers to Predict Chronic Kidney Disease and Hypertension at 3-Months Post-Cisplatin in Children
Date of Completion: April 10, 2024
Disclosure Updated Date: April 10, 2024

ASN
American Society of Nephrology
CJASN Kidney360 JASN

ASN Journal Disclosure Form

As per ASN journal policy, I have disclosed any financial relationship or commitment held by myself and/or my spouse/partner in the past 36 months as included below. I have listed my Current Employer below to indicate there is a relationship requiring disclosure. If no relationship exists, my Current Employer is not listed.

M. Zappitelli reports the following:
Employer: Toronto Hospital for Sick Children, University of Toronto; and Consultancy: Bioporto, Inc.

I understand that the information above will be published within the journal article, if accepted, and that failure to comply and/or to accurately and completely report the potential financial conflicts of interest could lead to the following: 1) Prior to publication, article rejection, or 2) Post-publication, sanctions ranging from, but not limited to, issuing a correction, reporting the inaccurate information to the authors' institution, banning authors from submitting work to ASN journals for varying lengths of time, and/or retraction of the published work.

Name: Michael Zappitelli
Manuscript ID: K360-2023-000809
Manuscript Title: Kidney and Blood Pressure Outcomes 11 Years After Acute Kidney Injury in Children: A Prospective Cohort Study
Date of Completion: November 29, 2023
Disclosure Updated Date: May 18, 2023
