# Supplementary material for: Tubular Injury Biomarkers to Predict CKD and Hypertension at 3 Months Post-Cisplatin in Children
Source: Kidney360. 2024 Apr 26;5(6):821–33. doi: 10.34067/KID.0000000000000448 (PMC11219117; doi:10.34067/KID.0000000000000448)
Supplement: Supplementary file 2 [file kidney360-5-821-s002.pdf]

Supplemental Material PDF

Supplemental Table 1. Urine AKI Biomarker Concentrations during Cisplatin Therapy in Participants with or without CKD or Hypertension at 3 Months Post-Cisplatin Treatment End.

Supplemental Table 2. Univariable and Multivariable Associations of Biomarkers with CKD and Hypertension at 3 Months Post-Cisplatin Treatment End

Supplemental Table 3: Univariable and Multivariable Associations of Biomarker Quartiles with CKD and Hypertension at 3 Months Post-Cisplatin Treatment End

Supplemental Table 4: Area Under the Receiver Operating Characteristics Curve for Sympercent Change in Urine AKI Biomarkers to Predict CKD and Hypertension at 3 Months Post-Cisplatin Treatment.

Supplemental Table 1. Urine AKI Biomarker Concentrations during Cisplatin Therapy in Participants with or without CKD or Hypertension at 3 Months Post-Cisplatin Treatment End.

Timepoint	3 Months	
	Median (IQR), n	Median (IQR), n
NGAL (ng/mg creatinine)		
	CKD	No CKD
EV Pre	15.77 (6.63-49.05), 48	14.06 (6.76-24.19), 65
EV Post	16.50 (6.23-65.25), 49	10.16 (5.35-23.60), 64
EV Discharge	**28.27 (9.56-76.20), 48**	**12.71 (5.01-34.36), 61**
LV Pre	13.33 (5.38-49.79), 37	15.38 (4.79-26.92), 52
LV Post	20.42 (8.07-61.16), 40	10.17 (5.49-23.76), 52
LV Discharge	15.81 (5.62-71.33), 38	12.58 (5.24-27.82), 47
	HTN	No HTN
EV Pre	16.62 (14.06-45.64), 17	14.54 (6.62-30.42), 103
EV Post	16.50 (5.94-21.98), 17	11.30 (5.29-28.81), 104
EV Discharge	34.36 (3.97-68.64), 15	15.05 (6.74-43.79)*, 101
LV Pre	12.30 (6.94-28.39), 12	15.11 (5.01-38.50), 86
LV Post	18.67 (5.70-33.04), 13	12.41 (5.28-26.78), 86
LV Discharge	23.89 (5.99-57.78), 12	15.39 (5.57-30.75), 83
KIM-1 (pg/mg creatinine)		
	CKD	No CKD
EV Pre	**542.82 (196.88-1101.27), 48**	**368.65 (152.65-705.31), 65**
EV Post	321.11 (185.19-856.47), 49	213.89 (145.20-492.03), 64
EV Discharge	**2415.76 (483.51-5099.19)*, 48**	**666.18 (257.24-3027.97)*, 61**
LV Pre	603.57 (257.46-1001.11), 37	442.83 (200.61-990.87), 52
LV Post	374.44 (189.04-1365.83), 40	242.34 (172.11-643.48), 52
LV Discharge	1848.79 (755.43-4689.72)*, 38	842.84 (341.54-3388.68)*, 47
	HTN	No HTN
EV Pre	248.47 (157.10-399.19), 17	457.53 (185.19-820.37), 103
EV Post	321.11 (185.19-1082.96), 17	230.55 (151.52-569.33), 104
EV Discharge	2674.11 (259.28-5211.95)*, 15	1468.02 (394.49-3303.32)*, 101
LV Pre	338.77 (125.98-3029.81), 12	504.90 (209.22-976.85), 86
LV Post	522.54 (192.90-5334.44), 13	351.06 (185.19-783.95), 86
LV Discharge	2429.28 (668.40-5360.03), 12	1049.26 (373.92-3388.68)*, 83
TIMP-2*IGFPB-7 ($(ng/ml)^2/1000$)		
	CKD	No CKD
EV Pre	0.014 (0.001-0.045), 51	0.015 (0.002-0.053), 66
EV Post	0.002 (0.0005-0.006), 50	0.001 (0.0005-0.003), 66
EV Discharge	0.007 (0.003-0.042)*, 48	0.006 (0.001-0.016)*, 63
LV Pre	0.012 (0.002-0.033), 39	0.026 (0.005-0.086), 52
LV Post	0.002 (0.001-0.010), 41	0.001 (0.0004-0.005), 52
LV Discharge	0.006 (0.002-0.033)*, 39	0.004 (0.001-0.021)*, 48
	HTN	No HTN
EV Pre	**0.003 (0.002-0.014), 17**	**0.020 (0.002-0.054), 107**

**EV Post**	0.001 (0.0003-0.002), 17	0.002 (0.001-0.004), 107
**EV Discharge**	0.007 (0.003-0.068)*, 16	0.006 (0.002-0.019)*, 102
**LV Pre**	0.004 (0.001-0.022), 12	0.015 (0.003-0.059), 88
**LV Post**	0.003 (0.001-0.009), 13	0.002 (0.001-0.006), 87
**LV Discharge**	0.004 (0.002-0.021)*, 12	0.004 (0.001-0.022)*, 84

Results stratified by time of cisplatin infusion. Bolded values represent a significant difference between Outcome and No Outcome groups by Mann-Whitney U Test: $P<0.05$.

*Biomarker levels are significantly different across the three time points (pre, post, discharge) within AKI or non-AKI groups by Skillings-Mack Test, $P<0.05$

Abbreviations: CKD: Chronic Kidney Injury, HTN: Hypertension, IQR, interquartile range, Pre: pre-cisplatin infusion, Post: post-cisplatin infusion, Discharge: near hospital discharge, EV: Early Visit, LV: Late Visit, IGFBP-7: insulin-like growth factor-binding protein 7, KIM-1: kidney injury molecule-1, NGAL: neutrophil gelatinase-associated lipocalin, TIMP-2: tissue inhibitor of metalloproteinase-2.

Supplementary Table 2: Univariable and Multivariable Associations of Biomarkers with CKD and Hypertension at 3 Months Post-Cisplatin Treatment End

Time of Collection	CKD		HTN	
	Unadjusted Odds Ratio (95% CI)	*Adjusted Odds Ratio (95% CI)*	*Unadjusted Odds Ratio (95% CI)*	*Adjusted Odds Ratio (95% CI)*
**NGAL (ng/mg creatinine)**				
**EV Pre-Infusion**	1.18 (0.88 - 1.60)	1.13 (0.77 - 1.67)	0.97 (0.66 - 1.44)	1.05 (0.65 - 1.71)
**EV Post-Infusion**	1.23 (0.94 - 1.61)	1.15 (0.82 - 1.64)	1.02 (0.71 - 1.48)	1.13 (0.69 - 1.88)
**EV Discharge**	**1.43 (1.07 - 1.92)**	**1.52 (1.04 - 2.26)**	1.09 (0.74 - 1.63)	1.09 (0.64 - 1.87)
**LV Pre-Infusion**	1.09 (0.81 - 1.50)	1.02 (0.68 - 1.55)	0.81 (0.52 - 1.26)	0.75 (0.44 - 1.32)
**LV Post-Infusion**	1.32 (0.95 - 1.86)	1.21 (0.79 - 1.87)	1.00 (0.65 - 1.56)	0.88 (0.52 - 1.52)
**LV Discharge**	1.16 (0.87 - 1.56)	1.01 (0.70 - 1.47)	1.21 (0.79 - 1.85)	0.95 (0.53 - 1.74)
**KIM-1 (pg/mg creatinine)**				
**EV Pre-Infusion**	**1.41 (1.01 - 1.97)**	**1.62 (1.11 - 2.38)**	0.68 (0.43 - 1.10)	0.65 (0.38 - 1.13)
**EV Post-Infusion**	1.07 (0.88 - 1.33)	1.04 (0.84 - 1.30)	1.26 (0.91 - 1.77)	1.17 (0.79 - 1.76)
**EV Discharge**	**1.47 (1.12 - 1.94)**	**1.39 (1.05 - 1.86)**	1.08 (0.76 - 1.56)	0.85 (0.58 - 1.26)
**LV Pre-Infusion**	1.28 (0.92 - 1.79)	1.23 (0.88 - 1.74)	0.94 (0.64 - 1.39)	0.83 (0.56 - 1.26)
**LV Post-Infusion**	1.24 (0.94 - 1.66)	1.18 (0.88 - 1.59)	1.46 (0.98 - 2.21)	1.17 (0.74 - 1.86)
**LV Discharge**	1.20 (0.91 - 1.59)	1.05 (0.78 - 1.43)	1.10 (0.73 - 1.67)	0.80 (0.53 - 1.24)
**TIMP-2*IGFBP-7 ((ng/ml)^2/1000)**				
**EV Pre-Infusion**	0.99 (0.84 - 1.17)	1.06 (0.89 - 1.28)	0.76 (0.60 - 0.97)	0.77 (0.58 - 1.04)
**EV Post-Infusion**	1.18 (0.92 - 1.54)	1.27 (0.95 - 1.71)	0.79 (0.54 - 1.18)	0.96 (0.60 - 1.56)
**EV Discharge**	1.18 (0.97 - 1.44)	1.16 (0.94 - 1.43)	1.08 (0.83 - 1.41)	0.96 (0.70 - 1.33)
**LV Pre-Infusion**	0.84 (0.69 - 1.03)	0.90 (0.73 - 1.12)	0.85 (0.65 - 1.11)	0.88 (0.63 - 1.23)
**LV Post-Infusion**	1.24 (0.96 - 1.63)	1.09 (0.82 - 1.47)	1.18 (0.81 - 1.71)	0.97 (0.60 - 1.56)

**LV Discharge**	1.13 (0.91 - 1.41)	1.03 (0.81 - 1.31)	1.04 (0.77 - 1.43)	0.72 (0.46 - 1.15)

Variables included in the multivariable model for CKD: sex, age at cisplatin treatment start <3 years, SCr-AKI during treatment, baseline estimated or measured GFR, and acyclovir use in 1 month before 3-month visit; Variables included in the adjusted model for hypertension: sex, age at cisplatin treatment start <3 years, and SCr-AKI during cisplatin treatment.

Supplemental Table 3: Univariable and Multivariable Associations of Biomarker Quartiles with CKD and Hypertension at 3 Months Post-Cisplatin Treatment End

			CKD		HTN	
	#	**Concentration (min-max)**	**Unadjusted OR (95% CI)**	**Adjusted OR (95% CI)**	**Unadjusted OR (95% CI)**	**Adjusted OR (95% CI)**
**NGAL (ng/mg creatinine)**						
**EV Pre-Infusion**						
**Q1**	**38**	**0.23 - 6.62**	**Reference**	**Reference**	**Reference**	**Reference**
**Q2**	**37**	**6.64 - 14.61**	0.76 (0.26 - 2.22)	0.81 (0.25 - 2.67)	0.75 (0.15 - 3.69)	0.57 (0.08 - 3.91)
**Q3**	**38**	**15.09 - 36.31**	0.56 (0.19 - 1.68)	0.71 (0.21 - 2.34)	1.16 (0.28 - 4.80)	1.85 (0.36 - 9.55)
**Q4**	**37**	**36.68 - 710.68**	1.67 (0.57 - 4.84)	1.6 (0.38 - 6.69)	1.41 (0.34 - 5.90)	1.28 (0.17 - 9.86)
**EV Post-Infusion**						
**Q1**	**37**	**0.55 - 5.57**	**Reference**	**Reference**	**Reference**	**Reference**
**Q2**	**37**	**5.68 - 12.29**	0.94 (0.31 - 2.93)	0.76 (0.23 - 2.55)	0.54 (0.09 - 3.19)	0.68 (0.09 - 5.06)
**Q3**	**37**	**12.45 - 28.7**	1.23 (0.43 - 3.54)	0.98 (0.29 - 3.32)	2.33 (0.62 - 8.72)	2.38 (0.46 - 12.39)
**Q4**	**37**	**28.91 - 665.46**	2.47 (0.83 - 7.39)	2.00 (0.50 - 7.94)	0.81 (0.16 - 3.96)	1.25 (0.16 - 9.72)
**EV Discharge**						
**Q1**	**37**	**0.61 - 6.57**	**Reference**	**Reference**	**Reference**	**Reference**
**Q2**	**36**	**6.74 - 15.48**	1.77 (0.55 - 5.66)	1.62 (0.46 - 5.72)	0.45 (0.08 - 2.65)	0.63 (0.10 - 3.96)
**Q3**	**37**	**15.8 - 54.70**	2.55 (0.84 - 7.72)	2.82 (0.79 - 10.08)	0.65 (0.13 - 3.17)	0.70 (0.10 - 3.76)
**Q4**	**36**	**55.29 - 1346.7**	**4.50 (1.49 - 13.56)**	**5.47 (1.23 - 23.84)**	1.97 (0.49 - 7.99)	2.92 (0.42 - 20.42)
**LV Pre-Infusion**						
**Q1**	**30**	**0.21 - 5.38**	**Reference**	**Reference**	**Reference**	**Reference**
**Q2**	**30**	**5.48 - 14.90**	1.35 (0.40 - 4.50)	1.23 (0.34 - 4.47)	3.16 (0.55 - 18.11)	2.06 (0.30 – 14.00)
**Q3**	**30**	**15.31 - 40.30**	0.58 (0.18 - 1.91)	0.71 (0.20 - 2.57)	2.18 (0.36 - 13.11)	2.88 (0.40 - 20.91)
**Q4**	**30**	**40.44 - 813.92**	1.83 (0.56 - 6.03)	1.74 (0.39 - 7.75)	0.57 (0.05 - 6.76)	0.53 (0.03 - 8.42)
**LV Post-Infusion**						
**Q1**	**31**	**0.85 - 5.29**	**Reference**	**Reference**	**Reference**	**Reference**
**Q2**	**31**	**5.32 - 12.23**	0.40 (0.11 - 1.48)	0.39 (0.10 - 1.63)	1.15 (0.21 - 6.35)	1.14 (0.17 - 7.82)

**Q3**	**31**	**12.58 - 32.86**	1.84 (0.58 - 5.87)	1.77 (0.46 - 6.82)	0.96 (0.18 - 5.24)	1.16 (0.17 - 7.99)
**Q4**	**30**	**33.04 - 879.57**	1.73 (0.53 - 5.72)	1.43 (0.32 - 6.27)	1.61 (0.32 - 8.12)	1.41 (0.17 - 11.57)
**LV Discharge**						
**Q1**	**29**	**0.56 - 5.67**	**Reference**	**Reference**	**Reference**	**Reference**
**Q2**	**29**	**5.81 - 16.23**	1.36 (0.42 - 4.4)	0.96 (0.26 - 3.51)	1.89 (0.29 - 12.51)	1.05 (0.12 - 9.35)
**Q3**	**29**	**17.34 - 35.16**	0.47 (0.13 - 1.69)	0.45 (0.11 - 1.88)	1.04 (0.14 - 8.04)	0.89 (0.86 - 9.31)
**Q4**	**29**	**36.43 - 1348.49**	**3.9 (1.06 - 14.39)**	2.89 (0.57 - 14.67)	3.53 (0.61 - 20.38)	1.64 (0.16 - 16.96)
**KIM-1 (pg/mg creatinine)**						
**EV Pre-Infusion**						
**Q1**	**38**	**21.30 - 185.12**	**Reference**	**Reference**	**Reference**	**Reference**
**Q2**	**37**	**185.19 - 394.69**	1.89 (0.62 - 5.77)	2.00 (0.58 - 6.93)	0.62 (0.16 - 2.40)	0.52 (0.11 - 2.52)
**Q3**	**38**	**396.67 - 844.68**	2.57 (0.86 - 7.62)	**3.92 (1.16 - 13.23)**	0.35 (0.08 - 1.48)	0.29 (0.05 - 1.52)
**Q4**	**37**	**857.12 - 21628.24**	**3.44 (1.13 - 10.43)**	**6.01 (1.69 - 21.44)**	0.45 (0.10 - 1.93)	0.54 (0.09 - 3.13)
**EV Post-Infusion**						
**Q1**	**37**	**0.57 - 154.58**	**Reference**	**Reference**	**Reference**	**Reference**
**Q2**	**37**	**157.83 - 235.87**	0.94 (0.31 - 2.83)	0.69 (0.21 - 2.27)	3.36 (0.62 - 18.19)	2.03 (0.32 - 12.78)
**Q3**	**37**	**243.59 - 618.95**	1.40 (0.49 - 4.02)	1.19 (0.39 - 3.63)	2.07 (0.35 - 12.27)	2.20 (0.31 - 15.77)
**Q4**	**37**	**619.88 - 41537.04**	2.13 (0.72 - 6.32)	1.64 (0.50 - 5.34)	2.92 (0.52 - 16.42)	1.89 (0.26 - 13.69)
**EV Discharge**						
**Q1**	**37**	**0.63 - 394.49**	**Reference**	**Reference**	**Reference**	**Reference**
**Q2**	**36**	**410.93 - 1523.86**	1.83 (0.60 - 5.59)	1.15 (0.33 - 4.02)	0.39 (0.07 - 2.16)	0.22 (0.03 - 1.50)
**Q3**	**37**	**1528.11 - 4037.06**	3.02 (0.99 - 9.19)	2.38 (0.73 - 7.77)	0.65 (0.14 - 3.02)	0.27 (0.04 - 1.74)
**Q4**	**36**	**4179.01 - 23320.99**	**3.15 (1.09 - 9.10)**	2.26 (0.71 - 7.18)	1.08 (0.28 - 4.21)	0.38 (0.07 – 2.00)
**LV Pre-Infusion**						
**Q1**	**30**	**0.56 - 192.90**	**Reference**	**Reference**	**Reference**	**Reference**
**Q2**	**30**	**201.85 – 507.00**	0.56 (0.17 - 1.90)	0.55 (0.15 – 2.00)	0.37 (0.06 - 2.09)	0.46 (0.06 - 3.37)
**Q3**	**30**	**530.99 - 1128.17**	1.73 (0.53 - 5.72)	1.50 (0.42 - 5.38)	0.17 (0.02 - 1.55)	0.06 (0.00 - 0.76)

**Q4**	**30**	**1148.89 - 65811.85**	1.18 (0.35 - 4.02)	0.98 (0.26 - 3.64)	0.99 (0.23 - 4.26)	0.62 (0.10 - 3.76)
**LV Post-Infusion**						
**Q1**	**39**	**0.63 - 185.19**	**Reference**	**Reference**	**Reference**	**Reference**
**Q2**	**23**	**192.90 - 350.46**	1.60 (0.48 - 5.31)	1.50 (0.42 - 5.29)	1.24 (0.19 - 8.29)	1.00 (0.12 - 8.58)
**Q3**	**31**	**351.67 - 923.93**	1.69 (0.56 - 5.11)	1.28 (0.38 - 4.31)	0.75 (0.12 - 4.84)	0.18 (0.02 - 1.85)
**Q4**	**30**	**956.91 - 51711.11**	2.44 (0.76 - 7.82)	1.88 (0.51 - 6.96)	3.11 (0.69 - 14.04)	1.04 (0.16 - 6.71)
**LV Discharge**						
**Q1**	**29**	**2.14 - 451.63**	**Reference**	**Reference**	**Reference**	**Reference**
**Q2**	**29**	**519 - 1343.19**	1.30 (0.39 - 4.34)	1.22 (0.35 - 4.34)	1.05 (0.14 - 8.08)	0.84 (0.09 - 7.54)
**Q3**	**29**	**1445.00 - 4082.78**	2.29 (0.67 - 7.84)	1.86 (0.48 - 7.31)	2.88 (0.50 - 16.48)	1.29 (0.17 - 9.48)
**Q4**	**29**	**4419.45 - 41388.89**	1.88 (0.55 - 6.39)	1.14 (0.29 - 4.41)	1.92 (0.29 - 12.72)	0.52 (0.05 - 5.10)
**TIMP-2*IGFBP-7 ((ng/ml)^2/1000)**						
**EV Pre-Infusion**						
**Q1**	**39**	**0.00 - 0.001**	**Reference**	**Reference**	**Reference**	**Reference**
**Q2**	**38**	**0.001 - 0.013**	1.00 (0.34 - 2.93)	1.22 (0.37 - 4.04)	1.60 (0.41 - 6.24)	2.83 (0.43 - 18.63)
**Q3**	**39**	**0.013 - 0.042**	0.92 (0.33 - 2.57)	1.43 (0.46 - 4.47)	1.14 (0.29 - 4.53)	3.19 (0.49 - 20.58)
**Q4**	**38**	**0.045 - 1.879**	0.96 (0.34 - 2.73)	1.48 (0.46 - 4.70)	1.00 (95% CI unable to be calculated)	1.00 (95% CI unable to be calculated)
**EV Post-Infusion**						
**Q1**	**39**	**0.000 – 0.000**	**Reference**	**Reference**	**Reference**	**Reference**
**Q2**	**38**	**0.001 - 0.002**	0.57 (0.20 - 1.62)	0.46 (0.14 - 1.49)	0.40 (0.09 - 1.70)	0.28 (0.05 - 1.48)
**Q3**	**38**	**0.002 - 0.004**	1.09 (0.38 - 3.15)	1.08 (0.33 - 3.56)	0.57 (0.15 - 2.19)	0.62 (0.12 - 3.14)
**Q4**	**38**	**0.004 - 1.056**	1.96 (0.70 - 5.48)	2.63 (0.83 - 8.27)	0.41 (0.10 - 1.77)	0.91 (0.17 - 4.94)
**EV Discharge**						
**Q1**	**37**	**0.000 - 0.002**	**Reference**	**Reference**	**Reference**	**Reference**
**Q2**	**37**	**0.002 - 0.007**	**3.64 (1.22 - 10.84)**	2.96 (0.95 - 9.21)	1.33 (0.27 - 6.53)	0.74 (0.13 - 4.18)
**Q3**	**37**	**0.007 - 0.032**	1.67 (0.55 - 5.01)	1.56 (0.49 - 9.21)	1.04 (0.19 - 5.62)	0.66 (0.10 - 4.34)
**Q4**	**37**	**0.034 - 0.711**	**3.11 (1.05 - 9.22)**	2.60 (0.82 - 8.27)	2.45 (0.55 - 10.96)	1.00 (0.18 - 5.64)

**LV Pre-Infusion**						
**Q1**	**31**	**0.000 - 0.002**	**Reference**	**Reference**	**Reference**	**Reference**
**Q2**	**30**	**0.002 - 0.012**	0.74 (0.22 - 2.54)	1.00 (0.26 - 3.77)	0.70 (0.15 - 3.34)	0.63 (0.11 - 3.74)
**Q3**	**31**	**0.119 - 0.052**	0.60 (0.19 - 1.94)	0.83 (0.23 - 2.95)	0.34 (0.06 - 1.91)	0.26 (0.04 - 1.83)
**Q4**	**30**	**0.054 - 0.654**	0.34 (0.10 - 1.11)	0.59 (0.15 - 2.27)	0.35 (0.06 – 2.00)	0.63 (0.09 - 4.32)
**LV Post-Infusion**						
**Q1**	**31**	**0.000 - 0.001**	**Reference**	**Reference**	**Reference**	**Reference**
**Q2**	**31**	**0.001 - 0.002**	1.00 (0.29 - 3.46)	1.15 (0.31 - 4.28)	1.25 (0.19 - 8.23)	1.14 (0.14 - 9.34)
**Q3**	**31**	**0.002 - 0.006**	2.17 (0.68 - 6.88)	1.50 (0.43 - 5.29)	1.82 (0.30 - 11.02)	0.71 (0.08 - 6.03)
**Q4**	**31**	**0.006 - 0.162**	2.60 (0.80 - 8.49)	1.69 (0.47 - 6.07)	1.90 (0.31 - 11.57)	0.90 (0.11 - 7.49)
**LV Discharge**						
**Q1**	**30**	**0.001 - 0.001**	**Reference**	**Reference**	**Reference**	**Reference**
**Q2**	**29**	**0.001 - 0.005**	**6.30 (1.69 - 23.53)**	**6.00 (1.52 - 23.66)**	7.20 (0.80- 64.89)	4.07 (0.40 - 41.09)
**Q3**	**30**	**0.006 - 0.030**	2.49 (0.68 - 9.19)	1.71 (0.42 - 6.93)	2.09 (0.18 - 24.61)	0.22 (0.01 - 5.57)
**Q4**	**29**	**0.030 - 0.577**	**4.40 (1.17 - 16.57)**	2.80 (0.68 - 11.57)	4.24 (0.41 - 44.27)	0.45 (0.02 - 10.57)

Variables included in the Adjusted model for CKD: sex, age at cisplatin treatment start <3 years, SCr-AKI during treatment, baseline estimated or measured GFR, and acyclovir use in 1 month before 3-month visit; Variables included in the adjusted model for hypertension: sex, age at cisplatin treatment start <3 years, and SCr-AKI during cisplatin treatment.

Supplemental Table 4. Area Under the Receiver Operating Characteristics Curve for Sympercent Change in Urine AKI Biomarkers to Predict CKD and Hypertension at 3 Months Post-Cisplatin Treatment.

Time of Collection	AUC (95% CI) to Predict 3-Month CKD[a]	AUC (95% CI) to Predict 3-Month HTN[b]
**NGAL (ng/mg creatinine)**		
**EV Post - Pre**	0.55 (0.44-0.66), 109	0.57 (0.42-0.71), 117
**EV DC - Pre**	0.57 (0.46-0.68), 104	0.59 (0.42-0.75), 111
**LV Post - Pre**	0.54 (0.40-0.67), 85	0.53 (0.33-0.72), 94
**LV DC - Pre**	0.54 (0.40-0.67), 78	0.56 (0.33-0.80), 90
**LV Post - EV Pre**	0.56 (0.44-0.69), 88	0.53 (0.36-0.70), 95
**LV DC - EV Pre**	0.46 (0.33-0.59), 81	0.62 (0.48-0.76), 91
**KIM-1 (pg/mg creatinine)**		
**EV Post - Pre**	0.50 (0.39-0.62), 109	**0.73 (0.60-0.87), 117**
**EV DC - Pre**	0.56 (0.45-0.67), 104	0.62 (0.42-0.82), 111
**LV Post - Pre**	0.43 (0.31-0.56), 85	0.68 (0.50-0.86), 94
**LV DC - Pre**	0.45 (0.32-0.58), 78	0.61 (0.40-0.83), 90
**LV Post - EV Pre**	0.54 (0.41-0.67), 88	**0.70 (0.52-0.89), 95**
**LV DC - EV Pre**	0.50 (0.37-0.63), 81	0.62 (0.42-0.82), 91
**TIMP-2*IGFBP-7 ((ng/ml)^2/1000)**		
**EV Post - Pre**	0.56 (0.45-0.67), 115	0.60 (0.46-0.75), 123
**EV DC - Pre**	0.54 (0.43-0.65), 110	**0.65 (0.51-0.80), 117**
**LV Post - Pre**	**0.66 (0.54-0.78), 88**	0.64 (0.49-0.79), 97
**LV DC - Pre**	**0.65 (0.53-0.77), 82**	0.60 (0.43-0.78), 93
**LV Post - EV Pre**	0.56 (0.44-0.68), 92	**0.72 (0.58-0.86), 99**
**LV DC - EV Pre**	0.55 (0.42-0.67), 86	**0.68 (0.54-0.82), 95**

Results stratified by time of cisplatin infusion. Bolded AUCs: 95% CI did not cross 0.5.
[a]For CKD analyses, 118 patients were included. N ranged between 78-116 depending on time point combinations.
[b]For HTN analyses, 125 patients were included. N ranged between 90-123 depending on time point combinations
Abbreviations: CKD: Chronic Kidney Injury, HTN: Hypertension, AUC: Area Under the Curve, Pre: pre-cisplatin infusion, Post: post-cisplatin infusion, DC: near hospital discharge, EV: Early Visit, LV: Late Visit, IGFBP-7: insulin-like growth factor-binding protein 7, KIM-1: kidney injury molecule-1, NGAL: neutrophil gelatinase-associated lipocalin, TIMP-2: tissue inhibitor of metalloproteinase-2.
